# Tapenade: Spatial Quantification of Mechanical and Genetic Fields in Dense 3D Organoids From Cell to Tissue Scale

**DOI:** 10.21769/BioProtoc.5823

**Published:** 2026-10-05

**Authors:** Alice Gros, Jules Vanaret, Sham Tlili, Léo Guignard

**Affiliations:** Aix Marseille Univ, CNRS IBDM (UMR 7288), Turing Centre for Living Systems, Marseille, France;

**Keywords:** 3D image analysis, Organoids, Nuclei segmentation, Quantitative analysis, Spatial correlation, Multiscale, Open-source

## Abstract

Whole-mount 3D imaging of multilayered biological tissues enables quantitative analysis of cell states and organization in their spatial context. However, extracting unbiased and meaningful quantitative information from dense, multilayered samples remains challenging due to imaging artifacts, increased density, and limited signal-to-noise ratio. Open source bioimage analysis workflows tailored to this type of analysis are scarce, and analysis bottlenecks like image curation or cell segmentation are seldom available without coding expertise. Here, we present a step-by-step computational protocol for the analysis of dense 3D organoid datasets using the Tapenade (Thorough Analysis PipEliNe for Advanced DEep imaging) workflow. Starting from multichannel image stacks, the protocol guides users through software installation, registration and fusion of multi-view datasets, preprocessing, and nuclei segmentation. It further details the generation of quantitative outputs, including morphometric measurements, deformation fields, and spatial correlation analyses. The workflow can be executed through open-source Python scripts or user-friendly Napari interfaces, allowing interactive parameter tuning and 3D visualization at each stage. This pipeline provides an accessible and modular framework for nonspecialist users to perform reproducible, multiscale quantitative analysis of 3D organoid images, while retaining flexibility for advanced users to customize individual steps.

Key features

• Multiscale quantitative analysis of dense 3D tissues: developed on thick whole-mount immunostained stem cell aggregates, Tapenade enables quantitative analysis from the cellular to the tissue scale.

• End-to-end pipeline: Tapenade provides graphical interfaces, notebooks, and step-by-step tutorials to streamline preprocessing and quantitative measurements, including morphometrics, deformation fields, and spatial correlations.

• Integrated visualization and modular analysis: Napari plugins support interactive parameter tuning and real-time exploration of large volumetric datasets.

• Scalability: Tapenade is compatible with standard bioimaging formats (e.g., TIFF, OME-TIFF, CZI, Zarr) and scalable to large 3D datasets through lazy loading and batch-processing strategies.

## Graphical overview


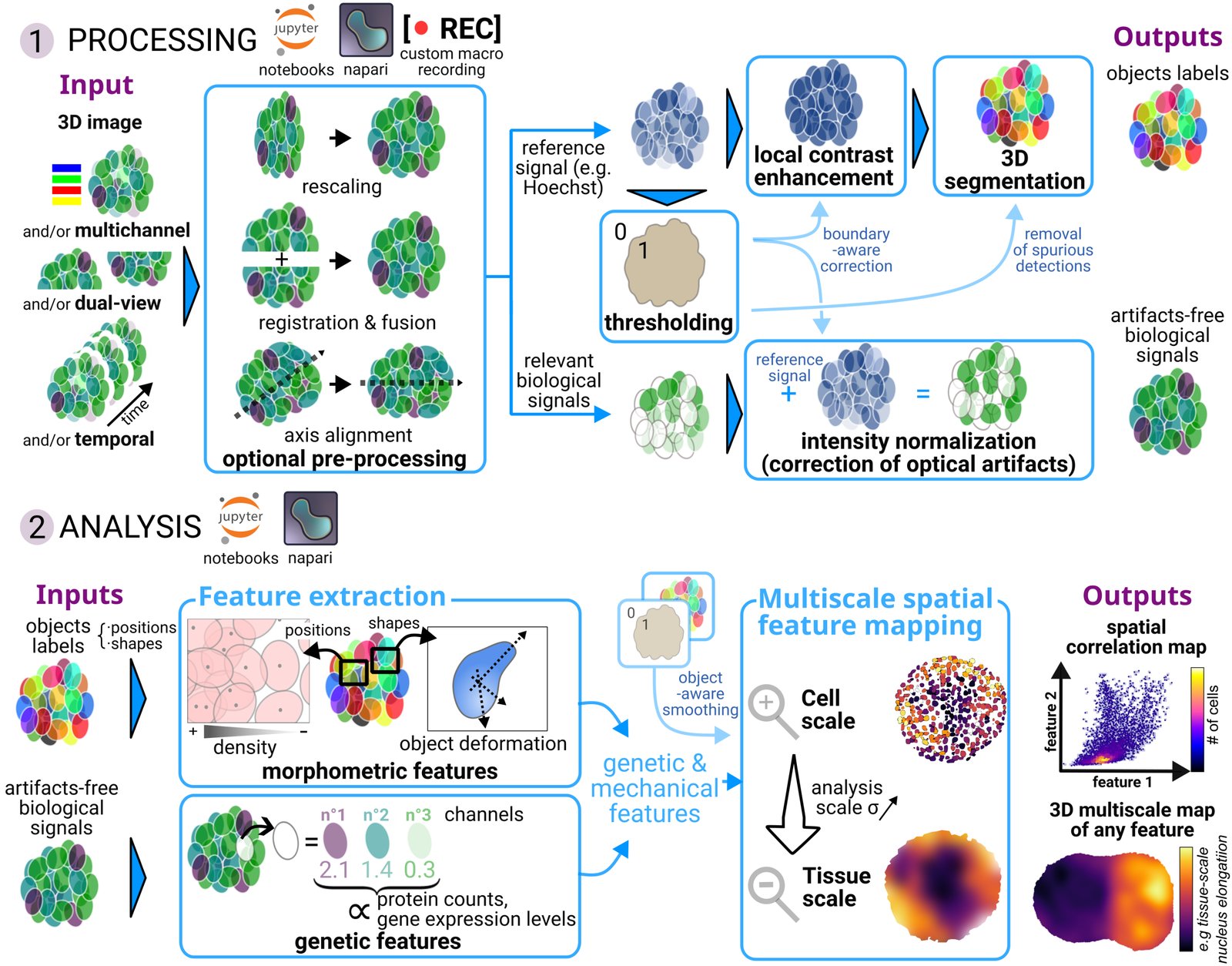


**Graphical overview of the Tapenade pipeline, separated into two modules: processing and analysis**

## Background

Organoids are self-organizing, three-dimensional (3D) cell cultures derived from stem or progenitor cells that recapitulate key structural and functional features of their organ of origin; tumoroids are their cancer-derived counterparts that preserve the characteristics of the original tumor. Owing to this ability to mimic native tissue in a controlled setting, 3D organoid and tumoroid models are increasingly used to study tissue organization, morphogenesis, and disease mechanisms in physiologically relevant contexts [1]. Whole-mount imaging enables the acquisition of volumetric datasets that preserve spatial organization, allowing the simultaneous analysis of tissue architecture, cell morphology, and gene expression at cellular resolution [2,3]. Quantifying these datasets, rather than describing them qualitatively, gives access to biological information that is otherwise inaccessible: metrics such as cell density, alignment, or object shapes reveal how tissues are mechanically and structurally organized. Cell and nuclear deformation, in particular, act as proxies for local mechanical stress that can drive morphogenetic flows [4] and influence cell differentiation [5]. Beyond individual metrics, correlating a gene expression signal with a mechanical or morphological property across the sample reveals how cell identity relates to tissue architecture in space, offering insight into tissue patterning during morphogenesis.

However, extracting quantitative information from these datasets remains technically challenging, particularly in dense and multilayered tissues, where optical artifacts, signal attenuation, and high cell density can compromise measurements and downstream analyses.

General-purpose open-source platforms such as Fiji [6] or QuPath [7] provide accessible solutions for image preprocessing, segmentation, and feature extraction, while integrating state-of-the-art deep learning methods such as StarDist [8] and Cellpose [9]. However, these tools remain limited for the analysis and exploration of complex 3D tissues, particularly when extensive customization or interactive visualization is required. In parallel, Python has become increasingly popular for bioimage analysis due to its mature scientific ecosystem (e.g., scikit-image [10], SciPy [11]) and its central role in the development of modern deep learning–based image analysis methods. Interactive visualization platforms such as Napari [12] further expanded the possibilities for exploring large multidimensional datasets through a plugin architecture similar to that of Fiji. Despite these advances, many Python-based workflows are not specifically optimized for dense 3D tissues, where voxel anisotropy, depth-dependent signal loss, and segmentation uncertainty can propagate errors into quantitative analyses. In addition, advanced workflows often require combining multiple tools and custom scripts, which can reduce reproducibility and limit accessibility for users without programming experience.

The Tapenade workflow was introduced in [13] as an integrated image analysis framework for 3D organoid imaging accessible to non-programmers. It combines multi-view registration, nuclei segmentation, correction of optical artifacts from gene expression signals, and multiscale quantitative analysis within a modular workflow specifically designed for dense 3D samples. The pipeline supports both notebook execution and interactive analysis through user-friendly Napari plugins, enabling parameter optimization, image exploration in 2D and 3D, and batch processing with reusable macros once optimal parameters have been established.

The protocol presented here builds on these developments and aims to complement the existing Tapenade documentation and tutorials with a practical step-by-step guide. In addition to describing the workflow, it provides recommendations for parameter tuning, data processing, and quantitative analysis to facilitate the use of Tapenade by non-expert users.

Compared with existing approaches, this protocol provides an end-to-end workflow from raw image stacks to quantitative outputs, ensuring consistency between preprocessing and downstream analyses. It supports multiscale analyses ranging from the cellular to the tissue scale, including morphometric measurements such as cell density, local alignment, deformation fields, and spatial correlations between distinct features, for example, genetic and mechanical properties. Its modular design also allows individual processing steps to be adapted or replaced depending on the imaging modality or experimental setup. Finally, the integration of interactive graphical interfaces facilitates iterative parameter optimization and data exploration, which is particularly valuable for large and complex 3D datasets.

Protocol performance is strongly influenced by image quality and resolution, particularly for segmentation, which requires approximately isotropic sampling and sufficiently resolved objects. Systematic segmentation errors or strong anisotropy along the axial dimension may introduce biases in downstream analyses. Multiscale analyses tolerate random, locally canceling segmentation errors, but hollow or cavity-rich architectures (e.g., lumens or epithelial monolayers surrounding large cavities) remain a current limitation: they fall outside the pipeline’s assumption of dense bulk tissue and would require dedicated masking.

Beyond organoid systems, this protocol can be applied to a wide range of tissues and modalities. It is particularly relevant for large, dense 3D specimens comprising multiple cell layers and typically exceeding 50 μm in thickness, such as organoids, spheroids, or tumoroids, but also embryos and explants. It is compatible with all 3D photonic imaging modalities, including confocal, two-photon, and light-sheet microscopy, in both live and fixed samples. More broadly, the pipeline provides a general framework for linking cell-scale measurements to tissue-scale organization, enabling quantitative studies of spatial patterning, including gene expression, mechanical deformation, and tissue organization in complex biological systems.

## Software and datasets

**Table:** 

Type	Software/dataset/resource	Version	Date	License	Access
Software	Tapenade (GitHub: GuignardLab/tapenade; PyPI: tapenade)	1.0.2	2026-05-14	MIT	free
Software	napari-tapenade-processing (GitHub: GuignardLab/napari-tapenade-processing; PyPI: napari-tapenade-processing)	1.0.2	2026-05-14	MIT	free
Software	napari-manual-registration (GitHub: GuignardLab/napari-manual-registration; PyPI: napari-manual-registration)	1.0.1	2026-05-14	BSD3	free
Software	napari-file2folder (GitHub: GuignardLab/napari-file2folder; PyPI: napari-file2folder)	1.0.2	2026-05-14	MIT	free
Software	napari-spatial-correlation-plotter (GitHub: GuignardLab/napari-spatial-correlation-plotter; PyPI: napari-spatial-correlation-plotter)	1.0.1	2026-05-14	MIT	free
Dataset	demo_data.zip (https://zenodo.org/records/15815474)	v2	2026-01-27	CC BY 4.0	free
Resource	environment.yml Anaconda file (Github: https://github.com/GuignardLab/tapenade/blob/main/environment.yml)		2026-08-03	MIT	free

We have tested the packages on Python versions ranging from 3.9 to 3.13, and on both Windows (versions 10 and 11) and Linux (Debian 12, Debian 13, and Ubuntu 22.04), with or without GPU. The pipeline was validated using an Intel i7 or i9 CPU, 64 GB RAM, and an NVIDIA GPU with 16 GB VRAM, such as Nvidia RTX Pro 2000 Blackwell, or NVIDIA GeForce RTX 3000 series. The minimum recommended is 16 GB RAM for large stacks. For StarDist3D segmentation, a CUDA-capable GPU (≥8 GB VRAM) is recommended, although not necessary. Without a local GPU, segmentation can be run for free via the ZeroCostDL4Mic Colab notebook. No GPU is required for the preprocessing, smoothing, correlation, or deformation analysis steps.

The installation instructions are provided below in section A. Though we will keep track of future updates of our main dependencies to make sure the installation instructions remain updated, we also provide an “environment.yml” file from a working Anaconda environment with Python version 12 and all packages installed (excluding GPU-dependent installs like Cellpose or StarDist). See section A below for installation instructions to use the “environment.yml” file.

## Procedure

The Tapenade package is organized as a modular workflow that includes registration and fusion, segmentation, correction of optical artifacts, spatial correlation analysis, and deformation tensor analysis; these steps can be run independently when needed.

As this protocol focuses only on computational modules of the Tapenade pipeline, spectral filtering as described in [13] is not included. Details of the experimental procedure can be found in [13], and an open-source script to apply spectral filtering is accessible in the Tapenade GitHub repository.

Section A: Package installation

Section B: Running the pipeline (Napari tutorial and links to Jupyter Notebooks)

Sections C, D, and D1: Preprocessing module of the Tapenade pipeline

Section C: Napari manual-registration plugin for registration and fusion of dual-view images

Section D: Napari-tapenade processing plugin for rescaling, axis alignment, thresholding, image normalization, …

Section D1: Tutorial on the batch processing tools (macros)

Sections E, F, and G: Analysis module of the Tapenade pipeline

Section E: Napari-correlation plotter plugin to display correlation maps of two signals (e.g., gene expression signals) and basic morphology features such as cell density.

Section F: Jupyter notebook (spatial_features_morphometrics_notebook.ipynb) for computing advanced 3D spatial features, including density gradients, volume fraction, and nuclei elongation; designed to explore the physical features of a given image and visualize tissue properties

Section G: Jupyter notebook (deformation_analysis_notebook.ipynb) for computing deformation fields of nuclei using inertia tensors and true strain tensors

**A. Installation of the package**

1. Install Anaconda (https://www.anaconda.com/download). Open Anaconda Prompt (find it in the search bar) or a terminal. Here, we use the Anaconda environment manager. Create a dedicated Python environment using Python 3.12 (if needed, the pipeline is compatible with Python versions 3.9–3.13).

*conda create -n env-tapenade python=3.12*

Alternatively, we provide an Anaconda environment file with pinned package versions for Python 3.12 (https://github.com/GuignardLab/tapenade/blob/main/environment.yml), which you can use to create the Anaconda environment **instead of the line above** using *conda create —file environment.yml*

Press y to continue installation. Once created, activate the environment by writing:

*conda activate env-tapenade*

Install the core packages, starting with the main library first.

*python -m pip install tapenade*

Then, install Napari for interactive 3D visualization.

*python -m pip install “napari[all]”*

2. (Optional) If you plan to perform dual-view registration and fusion, also install the specific dependencies.

*conda install -c conda-forge -c morpheme --strict-channel-priority vt*

*python -m pip install 3D-registration*

3. (Optional) Segmentation can be done using either CellPose-SAM [which gives good quality results for most datasets after making the pixel sizes isotropic (see section D) and local contrast enhancement without the need for retraining] or StarDist3D (for which we provide weights fine-tuned specifically for organoid data). We recommend starting with CellPose-SAM, which has higher versatility in terms of object morphology. StarDist3D is more suited for star-convex shapes such as round nuclei.

i. For CellPose-SAM, install pytorch first (https://pytorch.org/get-started/locally/). Then:

*python -m pip install cellpose*

ii. For StarDist3D, install tensorflow first (https://www.tensorflow.org/install), and then the StarDist package:

*python -m pip install stardist*

4. To download example datasets, download demo_data.zip from the following link: https://zenodo.org/records/17249972.

*Note: If you plan to only use Jupyter notebooks to run the pipeline, the notebooks will automatically download the test datasets and be written to process them if the user does not provide their own. This is done to illustrate a standard and controlled workflow.*

**B. How to run the pipeline**

The pipeline can be run using Napari or Jupyter Notebooks. This section presents

1. General recommendations for reproducibility

2. A basic Napari tutorial.

3. The Napari plugin napari-file2folder to load very large datasets and save them into tif sequences.

4. How to split channels or reorganize array dimensions to visualize multidimensional data correctly on Napari.

5. How to run the pipeline using Jupyter Notebooks as an alternative to Napari.

**1. General recommendations for reproducibility**

a. The single most important thing to track is the image scale. Beyond that, the workflow is robust to how you organize and name your files.

b. Track the voxel size during the pipeline for further quantitative measurements. The only metadata the workflow depends on is the image scale (voxel size), which the user can change during the registration and processing steps. Keep this information available for each dataset, either in the image metadata (if saved automatically) or in an accompanying text file. Throughout the workflow, scale is expressed in ZYX order with units of μm/pixel. For example, (0.5, 0.12, 0.12) μm/pixel denotes 0.5 μm spacing in Z and a pixel size of 0.12 μm in XY. If the scale changes during processing, record the scale of each output in an associated text file.

c. Save the parameter values used for preprocessing, segmentation, and smoothing alongside the outputs, for example, in a text file. For long pipelines, the “record macro” tool in the processing plugin does this automatically, saving each step and its parameters.

d. Save intermediate results such as masks and segmentations for reuse and validation. In both the Napari plugins and the notebooks, the default format is TIFF. We recommend non-experts to use it, as it can easily be read and written by most software. Note our terminology: a *mask* is a boolean array (1/True where the object is, 0/False elsewhere), whereas a *segmentation* is an instance segmentation in which each object carries a distinct label and the image is stored as unsigned integers. At any stage of the pipeline, the user can save intermediate results by using the “Save selected layers…” feature of Napari or by running dedicated saving commands in the Jupyter notebooks.

e. Naming convention is up to the user, because the user explicitly selects the input for each function, either in the code (notebooks) or via the buttons (Napari plugins). The plugins propose a convention that preserves the input name and appends the method, e.g., [FILENAME]_segmentation, but any scheme works.

f. The outputs and intermediate results of the pipeline are processed images, binary masks, segmentation labels, quantitative tables, and spatial maps that can be reproducibly reloaded for figure generation or validation on additional samples. This protocol was designed as a stepwise workflow that can be reproduced from installation to quantitative outputs without requiring users to assemble separate disconnected analysis tools.

**2. Napari tutorial**

*Note: Follow these steps and see Figure 1 to use the basic features of Napari.*

**Figure 1.**
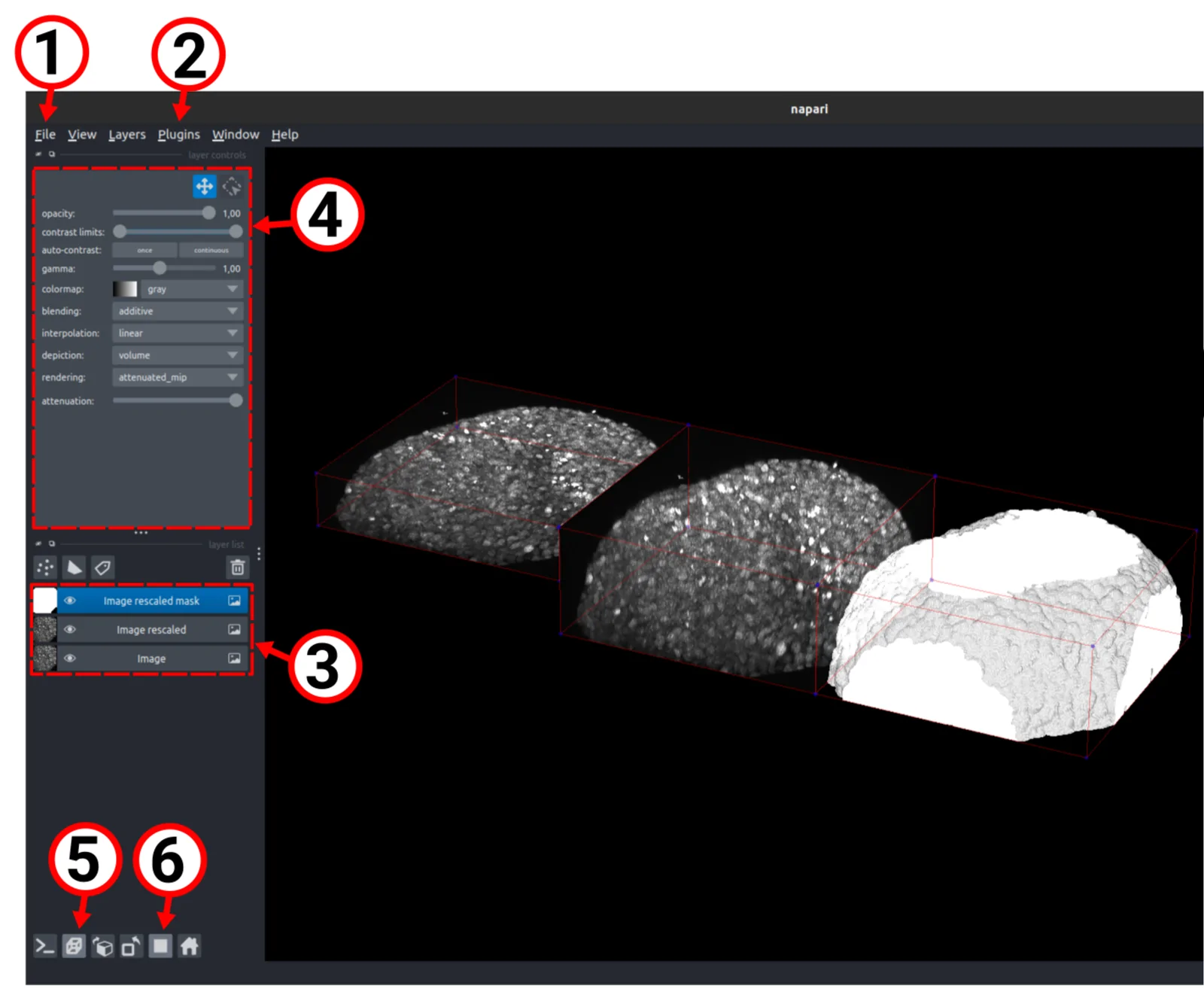
Step-by-step Napari tutorial. (1) Load images by drag-and-drop or *File* > *Open* (.tif, .ome-tiff, .nd2, .lsm, .zarr, .ome-zarr, .czi). Save via *File* > *Save Selected Layer*. (2) Plugin menu to install and open plugins as side widgets. (3) Layer list showing loaded images as layers (4) Adjustable per-layer parameters: contrast limits, colormap, opacity, blending mode. (5) Toggle 2D/3D view. (6) Toggle grid view. Pixel values shown at the bottom left.

To open Napari in the environment where Tapenade is installed:

*conda activate env-tapenade*

*napari*

① First, load your images in Napari. You can drag and drop them from your file explorer to the Napari viewer or open them using the *File* > *Open files*... menu. Compatible file formats are .tif, .ome-tiff, .nd2, .lsm, .zarr, .ome-zarr, and .czi. If you wish to save some images for future steps, use *File* > *Save Selected Layer*.

② The *Plugin* menu allows you to install new plugins or open the ones you have already installed. Plugins open as a widget on the side; you can open multiple at the same time.

③ The image you have loaded will be displayed as an individual layer in the *Layer List*. They can be clicked on to reveal a set of visual parameters (see B1e) that can be adjusted. By double-clicking on a layer name, you can change it. Right-clicking a layer will give you several options. The little eye icon next to the layer name can be clicked to hide the layer.

④ Adjust visual parameters for each layer, like the contrast limits, the colormap, the opacity, the blending mode, etc.

⑤ To switch between 2D and 3D views, click on *Toggle 2D/3D view* (it resembles a square when in 2D mode, or a cube when in 3D mode).

⑥ Toggle the grid view (as shown in the example image) by clicking on *Toggle grid mode*. By right-clicking the button, you can parametrize the grid view (e.g., number of columns, number of rows, etc.). Pixel values are indicated on the bottom left below buttons 5 and 6 when a layer is selected.

**3. Save large files into individual slices or timepoints with napari-file2folder (lazy loading)**

**Input:** The path to an n-dimensional file (e.g., TIFF, OME-TIFF, CZI, Zarr, nd2), potentially not fitting entirely in memory.

**Output:** A folder containing all elements of the input file along a given axis, saved as separate files.

If the image is too large to fit in your computer memory, use the plugin napari-file2folder (see Figure 2) to split your image into separate .tif files. Click on the *Plugins* menu to select and open the plugin *Save multidimensional file as a folder of .tif*.

**Figure 2.**
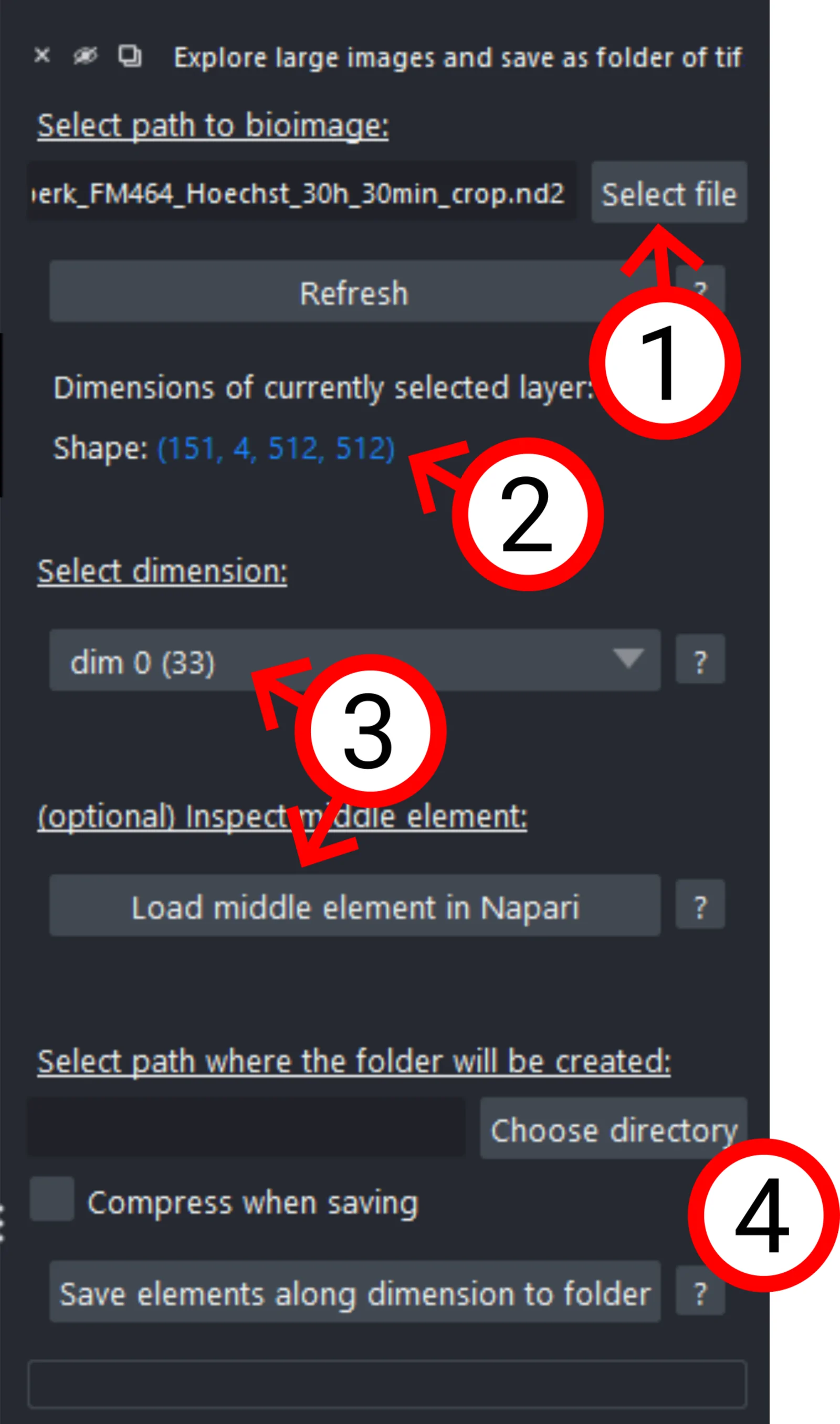
Step-by-step tutorial of napari-file2folder (1) Select the path to your bioimage (Bio-Formats such as .czi, .nd2, .lsm supported). (2) Array shape displayed below as TZCYX. (3) Choose the dimension to inspect its midpoint element (e.g., the middle channel or the midplane). (4) Save each element as a separate .tif in a chosen folder (e.g., splitting a stack into individual channel files).

① Select the path to your bioimage (bioformats like .czi, .nd2, and .lsm are supported).

② The shape of the array is displayed below under the format TZCYX.

③ Choose the dimension along which to inspect the midpoint element (this gives a preview of the type of images that will be saved). For instance, if you select the channel dimension and click *Load middle element*, this will display the middle channel on Napari.

④ You can then choose to save each element as a separate .tif file in the provided folder. For example, if you choose the channel dimension, this will save a folder containing the separated channels as individual files. Select the path where the folder will be created (click on *Choose directory*), then select *Save elements along dimension to folder.*

**4. Change the array shape using the widget *Re-organize array dimensions***

**Input:** An image, binary mask, or a segmentation.

**Output:** An image, binary mask, or a segmentation, whose axes have been swapped to fit the shape specified.

If your array fits in memory, you can drop a Hyperstack in the Napari window. For visualization purposes, you can choose to split the array into separate channels (as separate layers) to visualize them next to each other. For this, use the plugin *Tapenade Processing* and its widget *Re-organize array dimensions.* The widget also allows you to rearrange the image dimensions if they are not correctly interpreted (e.g., channels, time, or Z are misassigned) and the visualization is not correct.

① Drag and drop your image, select your array, and the dimensions will be displayed with T (time), C (channels), Z, Y, and X.

② You can choose to re-assign dimensions if necessary, using the dropdown menus, so they match the actual data structure, and select *Run function* to create a new array on Napari with the new dimensions.

③ To visualize individual channels, select *Separate channels* and *Run function*.

**Critical:** As the rest of the pipeline works in the convention ZYX for the spatial ordering (with Z the depth axis), make sure to reshape your datasets according to this convention.

**5. Run the pipeline using Jupyter notebooks**

① From the GitHub page, download the notebooks available: https://github.com/GuignardLab/tapenade/tree/main/src/tapenade/notebooks

For example, they can be run in Jupyter. To open one from the command line, write in your environment:

jupyter notebook

② Run cells one by one by pressing *Shift + Enter* or the *Run* button. Replace … signs by the paths to your data as a Python string. On Windows, write raw strings to avoid issues (note the “r” character at the beginning):

path = r”folder\image.tif”

③ Read the text cells of the notebook carefully, as they provide guidance on how to best apply the workflow to your data. You can also read the documentation and detailed parameters by writing help(function) in a new cell. Note that modern code edition software allows you to go to the function definition to see its documentation (e.g., in VSCode, right-click on the function and select *Go to definition)*.

④ Some notebooks will declare a *display_in_napari* variable at the beginning that you can set to *True* to visually inspect the results of each function in a Napari window and adjust the parameters. If you choose to use it, keep the Napari window open during the workflow.

**Warning**: The notebooks work in 3D and on the ZYX convention.

*Notes:*

*1. To apply to time sequences, use the Napari plugin napari-file2folder to split movies into frames saved as individual .tifs. You can then either apply the processing by running the code independently for each frame or use the macro recording feature of the Napari-Tapenade-processing plugin to apply your custom workflow in batch to the whole folder.*

*2. Most notebooks automatically download test datasets and are designed to process them if the user does not provide their own. This serves as an example of a standard, controlled workflow.*

## Processing modules

**C. Dual-view registration**

**Input:** A pair of images acquired from the same biological sample.

**Output:** An image obtained by fusing the two input images such that the parts they have in common correctly overlap.

Use the registration plugin to manually align two views of the same sample, typically acquired using dual-view or multi-angle microscopy systems. The plugin provides two complementary workflows:

1. Registration by manually selecting explicit transformation parameters.

2. Registration by annotating matching salient landmarks.

And how to proceed after registration:

3. Fusion of the registered views.

4. Saving transformation parameters once validated.

5. Loading and applying previously computed transformation parameters to another image (for example, another channel).

The image being registered is called floating view; the other view stays fixed and is called reference view.

Recommendations before starting the registration workflow:

a. The input data are the two 3D image datasets corresponding to different views of the same sample.

b. Image layers must contain integer or floating-point intensity values.

c. Both datasets must have identical dimensions and voxel sizes.

d. Recommended file formats include .tif, .tiff, or other formats supported by Napari.

e. If metadata is available, store voxel size information in μm/pixel in ZYX order.

*Note: Though Napari can compensate for voxel anisotropy during visualization, we suggest resampling anisotropic datasets to isotropic voxel size before registration to improve alignment accuracy and ensure physical interpretability in downstream analysis steps.*

**1. Registration by manually selecting explicit transformation parameters**

Use this workflow and Figure 3 to manually align the floating image to the reference image using rigid translations and rotations with real-time visual feedback.

**Figure 3.**
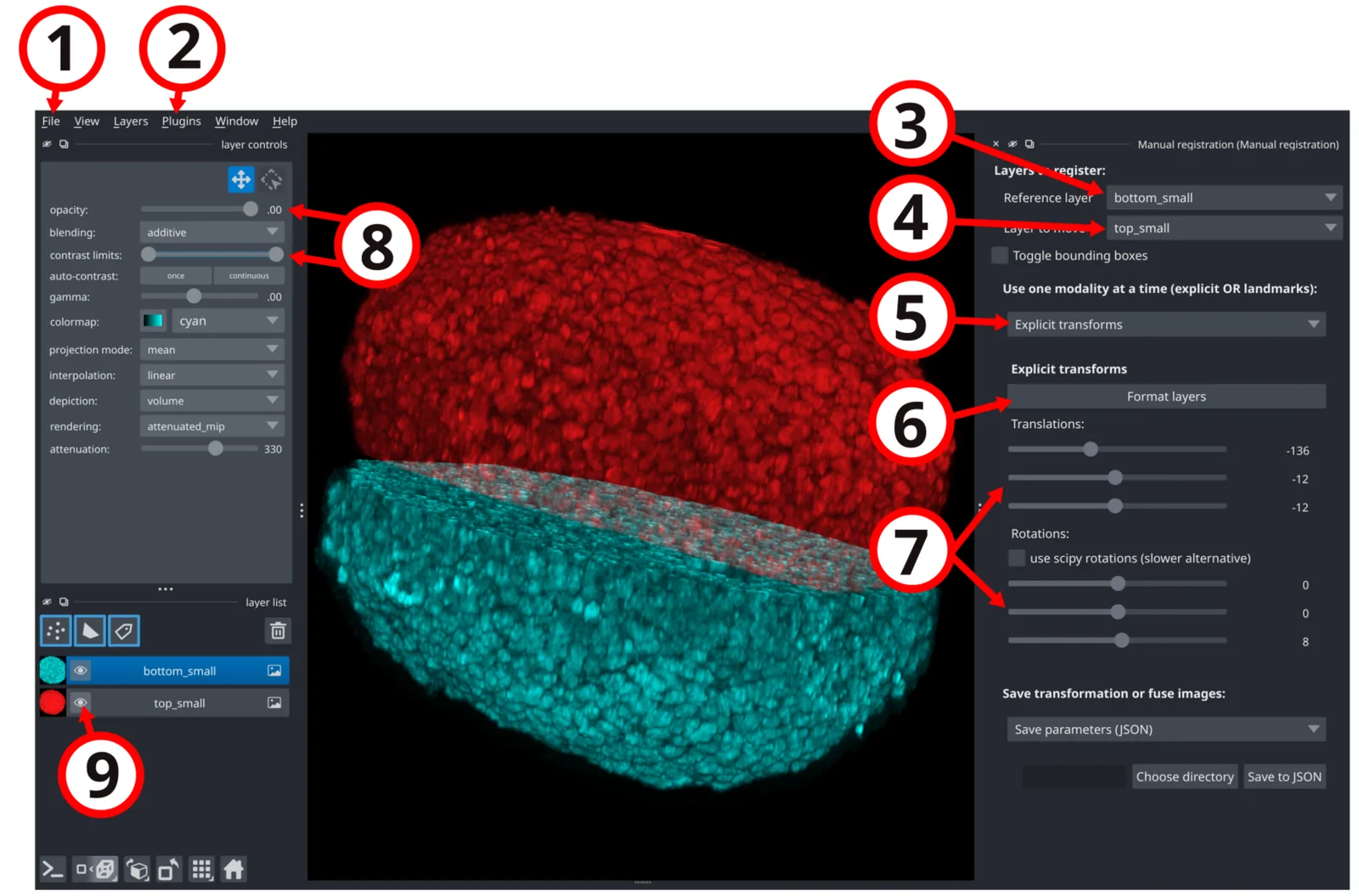
Step-by-step tutorial of Napari manual-registration using the sliders to manually specify translations and rotations. (1) Load the reference and floating images into Napari by drag-and-drop or *File* → *Open files*. (2) Open the plugin via *Plugins* → *Manual Registration*. (3) Set the fixed image in the *Reference* layer combo box. (4) Set the moving image in the *Floating* layer combo box. (5) Choose *Explicit transforms* (6) Optionally click *Format layers* to auto-optimize visualization. (7) Use the *Translations* and *Rotations* sliders to move and rotate the floating image along the three spatial axes, starting with coarse rotational alignment before refining translations until it overlaps the reference. (8) Adjust layer opacity and contrast. (9) Toggle layers with the eye icon and rotate the 3D camera to inspect alignment from multiple angles, combining 2D slice and 3D views for accuracy.

① Load the reference and floating images into Napari by dragging and dropping the files into the viewer or by selecting *File* → *Open files…*.

② Open the plugin by selecting *Plugins* → *Manual Registration*.

③ Select the fixed image from the *Reference layer* combo box

④ Select the moving image from the *Floating layer* combo box.

⑤ Select *Explicit transforms* from the workflow drop-down menu.

⑥ Optionally click *Format layers* to automatically optimize visualization settings.

⑦ Adjust the *Translations* sliders to move the floating image along the X, Y, and Z axes. Adjust the *Rotations* sliders to rotate the floating image around the three spatial axes. Begin by performing coarse rotational alignment before refining translations. Continue adjusting translations and rotations until the floating image visually overlaps the reference image.

⑧ Modify layer opacity and contrast in the Napari layer properties panel to improve visualization of the overlap.

⑨ Hide or display layers using the eye icon in the layer list as needed. Rotate the 3D camera view to inspect the alignment from multiple viewing angles. Combining 2D slice inspection with 3D visualization often improves registration accuracy.

**2. Registration by annotating matching salient landmarks**

Use this workflow and Figure 4 when the two views contain identifiable structures (keypoints) that can be matched manually, such as bright dead cells, lumens, or any characteristic tissue structure.

**Figure 4.**
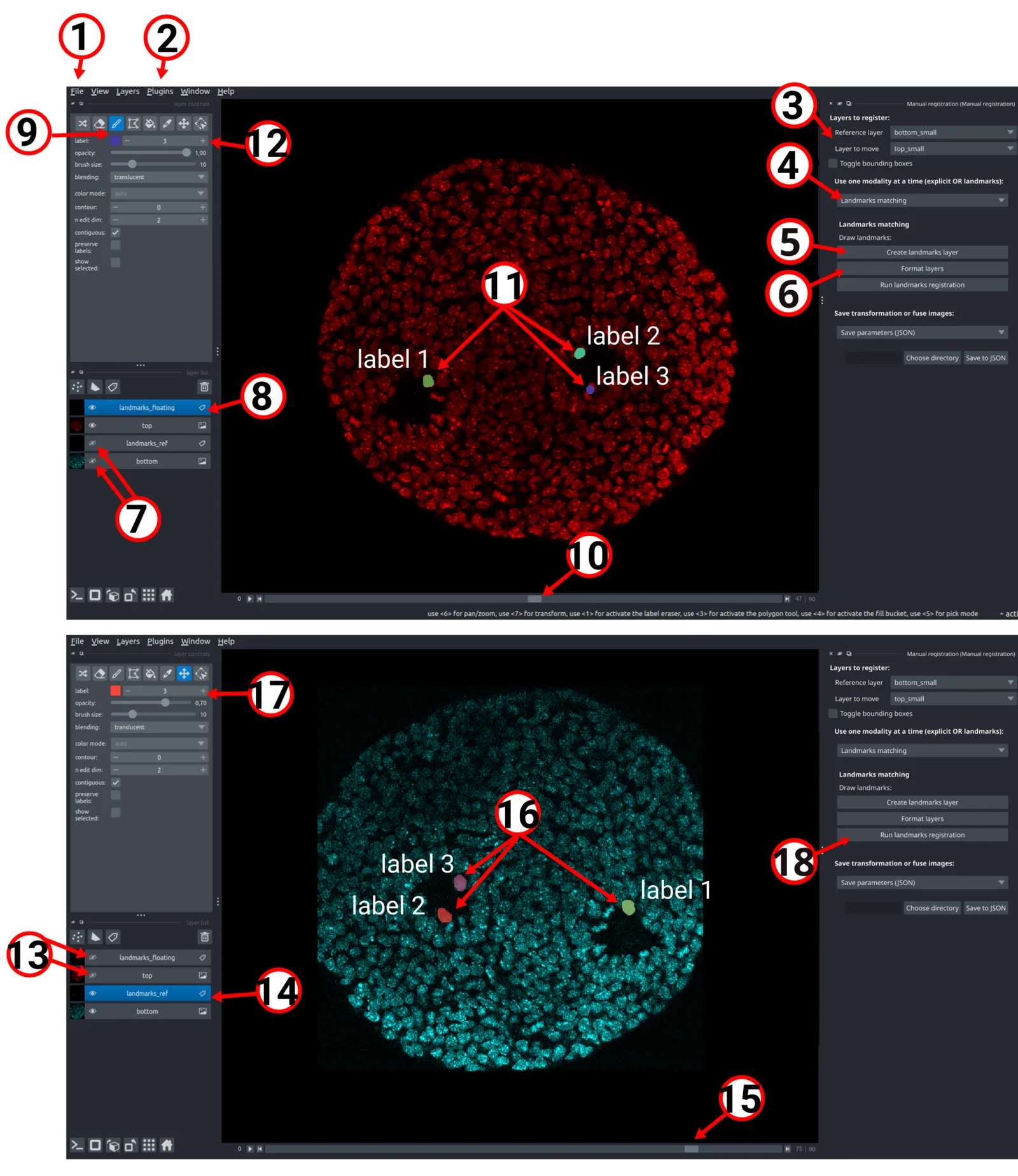
Step-by-step tutorial of napari-manual-registration using salient landmarks. (1) Load the reference and floating images into Napari by drag-and-drop or *File* → *Open files*. (2) Open the plugin via *Plugins* → *Manual Registration*. (3) Set the fixed image in the *Reference* layer combo box. (4) Set the moving image in the *Floating* layer combo box. (5) Choose *Landmarks matching*, then *Create landmarks* layers to generate the landmarks_reference and landmarks_floating label layers. (6) Optionally click *Format layers* for landmarks matching to auto-optimize visualization. (7) Hide the reference image and reference landmark layers with the eye icon. (8) Select landmarks_floating and adjust its opacity, contrast, and colormap for visualization. (9) Activate the paint brush. (10) Navigate the z-slices with the slider at the bottom of the viewer. (11) Draw a blob on a salient structure in the floating image; shape is unimportant, as the plugin uses the center of mass. (12) Increment the label value with the + button and repeat until all landmarks are annotated (minimum three). (13) Hide the floating layers and show the reference image and reference landmark layers. (14) Select landmarks_reference. (15) Go to the z-slice of the first floating landmark. (16) Draw a blob on the corresponding structure in the reference image. (17) Increment the label value after each annotation and repeat for all landmarks, annotating in the same order in both layers to preserve label correspondence. (18) Click *Run landmark registration* to compute the rigid transformation, then inspect the alignment in 2D and 3D.

① Load the reference and floating images into Napari by dragging and dropping the files into the viewer or by selecting *File* → *Open files…*.

② Open the plugin by selecting *Plugins* → *Manual Registration*.

③ Select the fixed image from the *Reference layer* combo box

④ Select the moving image from the *Floating layer* combo box.

⑤ Select *Landmarks matching* from the workflow drop-down menu. Click *Create landmarks layers* to generate two new label layers:

landmarks_reference

landmarks_floating

⑥ Optionally, click *Format layers for landmarks matching* to automatically optimize the visualization settings.

⑦ Hide the reference image and reference landmark layers by clicking the eye icon in the layer list.

⑧ Select the landmarks_floating layer. Adjust the opacity, contrast, and colormap settings in the Napari layer properties panel manually to help with visualization.

⑨ Activate the paint brush tool by clicking *Activate the paint brush*.

⑩ Navigate through the z-slices using the slider located at the bottom of the Napari viewer.

⑪ Identify a salient structure in the floating image and draw a blob around or on it. The exact blob shape is not important because the plugin uses the center of mass of the annotated region.

⑫ Increase the label value by clicking the + button in the label layer controls. Repeat steps C2i–l until all landmarks are annotated in the floating view (minimum three landmarks are necessary).

⑬ Hide the floating layers and display the reference image and reference landmark layers.

⑭ Select the landmarks_reference layer.

⑮Navigate to the z-slice corresponding to the first landmark identified in the floating image.

⑯ Draw a blob around the corresponding structure in the reference image.

⑰ Increase the label value after each landmark annotation. Repeat steps C2p–q until all corresponding landmarks are annotated. Annotate landmarks in the same order in both layers to preserve correspondence between labels.

⑱ Click *Run landmark registration* to compute the rigid transformation aligning the floating image to the reference image.

Inspect the resulting alignment in both 2D and 3D views.

*Note: If the alignment is unsatisfactory, add additional landmarks or refine landmark placement before running the registration again.*

**3. After registration, fuse registered views and/or save the registration parameters**

Once the correct registration parameters are found, you can apply the transformation and fuse the two views (Figure 5), and/or you can save the registration parameters into a .json file (Figure 6) to keep them for other channels, for example.

**Figure 5.**
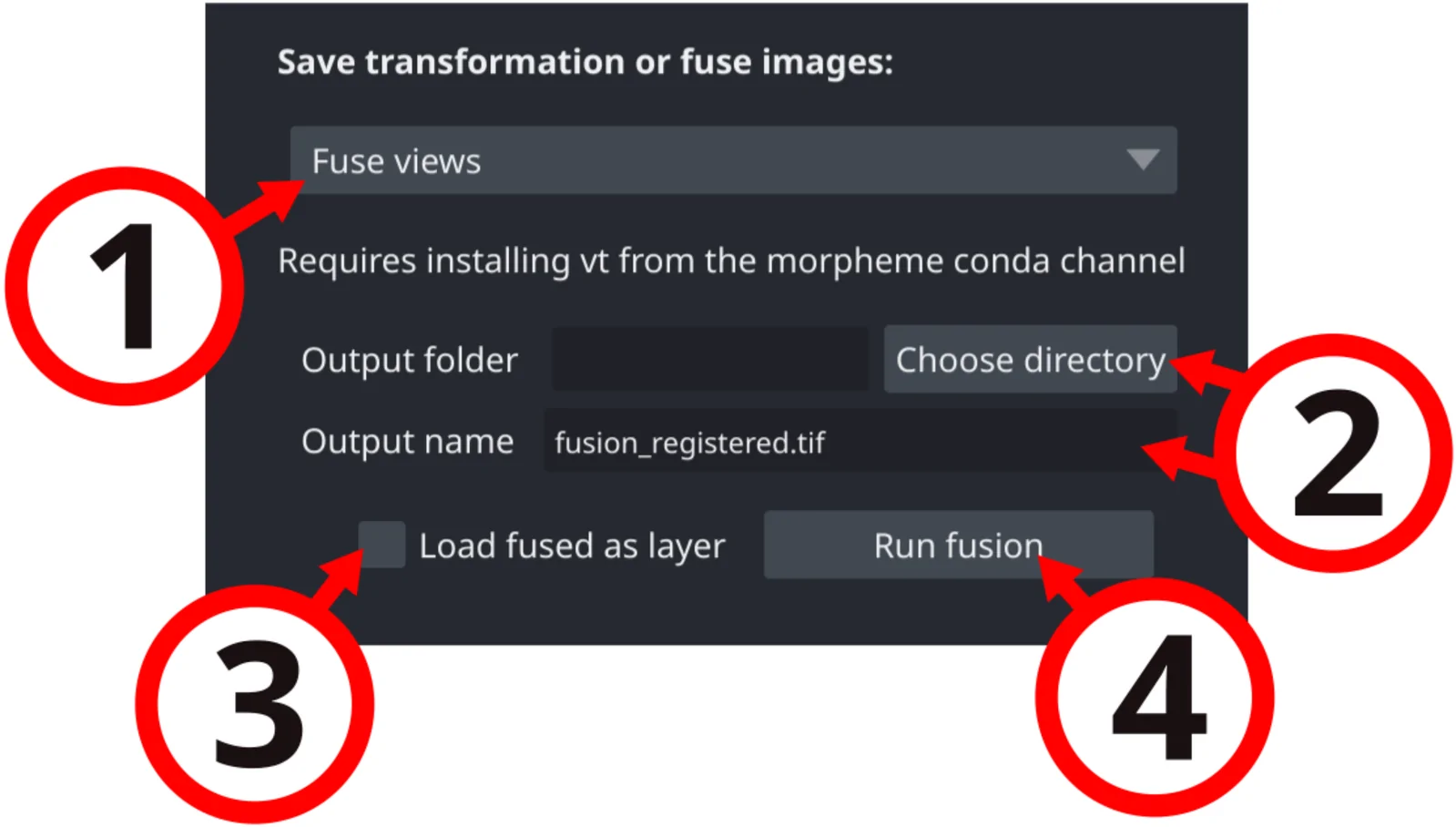
Step-by-step tutorial of Napari manual-registration to fuse two views after registration. (1) Choose *Fuse views* from the workflow drop-down. (2) Click *Choose directory* to set the output folder and enter a name in the *Output* name field. (3) Optionally enable *Load fused as layer* to display the fused image after fusion. (4) Click *Run fusion*, then inspect the result to confirm both views are correctly aligned.

① Select *Fuse views* from the workflow drop-down menu.

② Click *Choose directory* and select the output folder. Enter the desired output filename in the *Output name* field.

③ Optionally enable *Load fused as layer* to automatically display the fused image in Napari after fusion.

④ Click *Run fusion* to generate the fused image. Inspect the fused image to confirm that both views are correctly aligned.

**Warning**: Fusion quality strongly depends on the accuracy of the registration step. Verify alignment carefully before exporting the fused dataset.

**4. Save transformation parameters to a JSON file**

① Once the registration is satisfactory, select *Save Parameters (JSON)* from the workflow drop-down menu.

② Click *Choose directory* and select the output folder.

③ Click *Save to JSON* to export the rigid transformation parameters.

**Figure 6.**
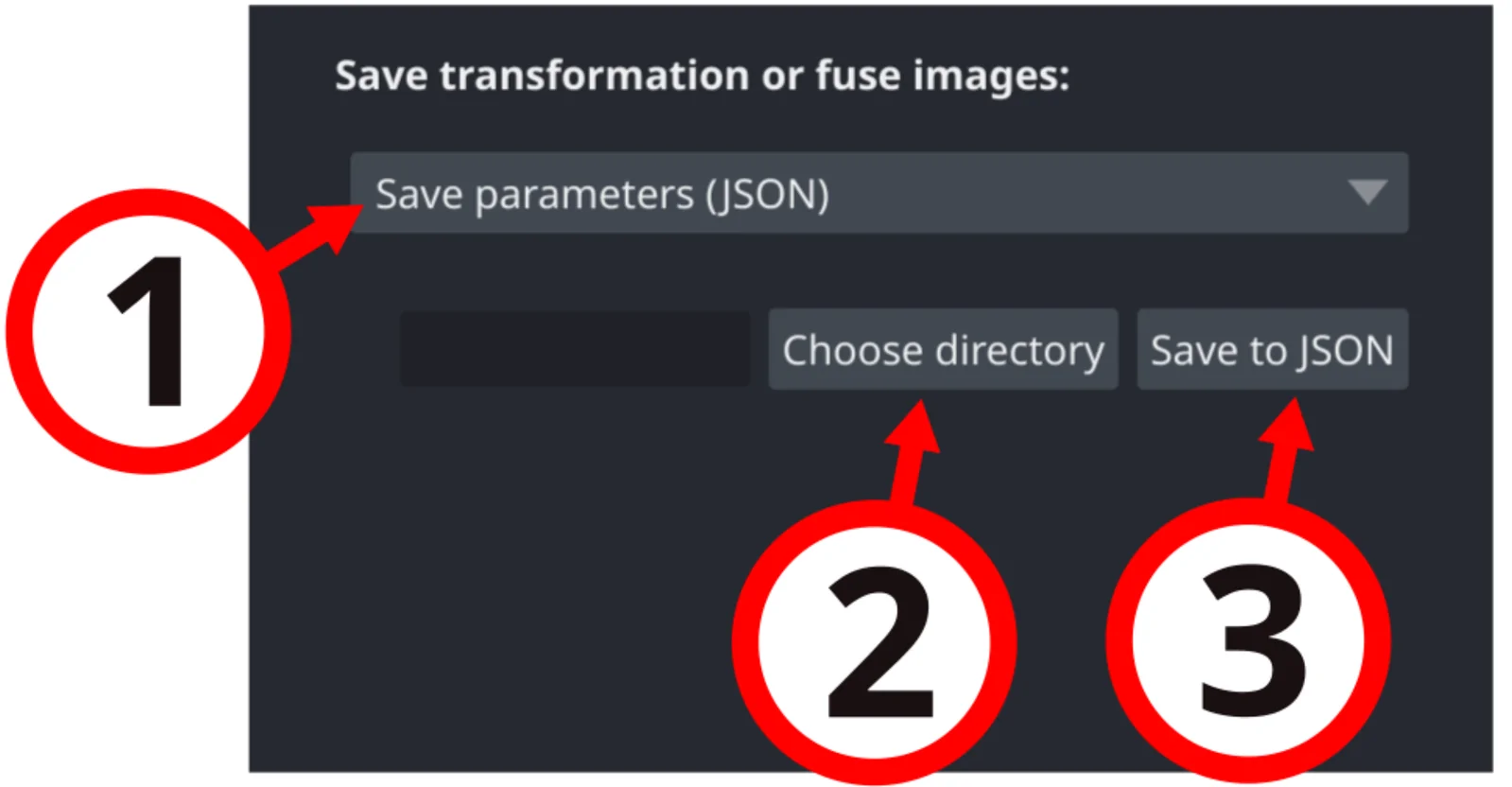
Step-by-step tutorial of Napari manual-registration to save the registration parameters

**5. Load a JSON file with transformation parameters and apply to loaded views**

Use this mode to apply a previously saved transformation (from section C) to the currently selected “Layer to move.” This can be useful if you have multichannel images and you want to apply the same registration transform to all channels. To that end, compute the registration using the sliders or the landmarks, save the transform parameters as a JSON file, and apply it to each channel iteratively. The same principle can be used for temporal images by applying the same registration transform to each time frame sequentially.

**Warning**: The plugin supports 3D datasets organized in ZYX (with Z the depth dimension) axis convention.

*Note: To know whether your movie has the correct order of dimensions, use the plugin napari-tapenade-processing and its widget* Re-organize your array dimensions. *Dimensions of the array as read by Napari will be indicated, and you can invert dimensions (for example, invert T and Z) to match the correct shape of your image.*

If you want to apply the registration to all frames of a movie, or to a multichannel dataset with a large amount of channels, you might want to use automatic registration in a script, e.g., via our registration Jupyter Notebook to automatically iterate through time frames or channels instead of doing it manually in Napari.

**D. Processing**

From a given set of raw images, segmented object instances, and object masks, the plugin napari-tapenade-processing allows the user to quickly run all preprocessing functions from our main pipeline with custom parameters while being able to see and interact with the result of each step. For large datasets that are cumbersome to manipulate or cannot be loaded in Napari, the plugin provides a macro recording feature: users can experiment and design their own pipeline on a smaller subset of the dataset, then run it on the full dataset automatically (section D1).

The Jupyter Notebooks preprocessing_notebook.ipynb, segmentation_notebook_stardist.ipynb, and segmentation_notebook_cellpose.ipynb include the same functions as the plugin for processing and segmentation.

This section presents:

1. An overview of the plugin structure

2. Change layer voxel size

3. Compute a mask from an image

4. Crop layers using a mask

5. Apply local contrast enhancement

6. Segment the nuclei

7. Remove the labels outside of the mask

8. Apply intensity renormalization

9. Align layers from the mask major axis

10. Apply masked Gaussian smoothing

**1. Understanding the structure of the plugin**

The plugin is composed of three tabs. The first tab is dedicated to the choice of preprocessing functions, the second tab is dedicated to the macro recording feature, and the third tab is dedicated to advanced parameters. The preprocessing tab is composed of the following elements (see Figure 7):

① A combo box to select the preprocessing function to apply from a list.

② A set of boxes that allows you to select the layers on which to apply the function. If a function does not require a specific layer, the combo box will be greyed out. *Image* layers correspond to integer or float data, *Labels* layers correspond to integer data and represent segmented object instances, and *Mask* layers correspond to Boolean data and usually represent the sample’s large-scale mask (inside/outside). All layers must have data of the same shape (same number of dimensions and same dimensions). Layers can be 3D or 3D+time, respectively, with the ZYX or TZYX order.

③ If a layer does not appear in the combo box, but is present in the *Layer* list, you can click on *Refresh* to update the list of layers.

④ A set of parameters that you can tune to adjust the function’s behavior. The parameters are specific to each function. In case of doubt, you can click on the [?] button next to the widget to get a tooltip with a description.

⑤ A *Run function* button to apply the function with the current parameters to the previously selected layers.

The third tab contains a checkbox, *New layers overwrite previous ones*, determining whether the output of the preprocessing functions should be saved as new layers or overwrite the previous ones that were used as input. This can be useful to save memory when you do not need to compare the input and output of a function.

**Figure 7.**
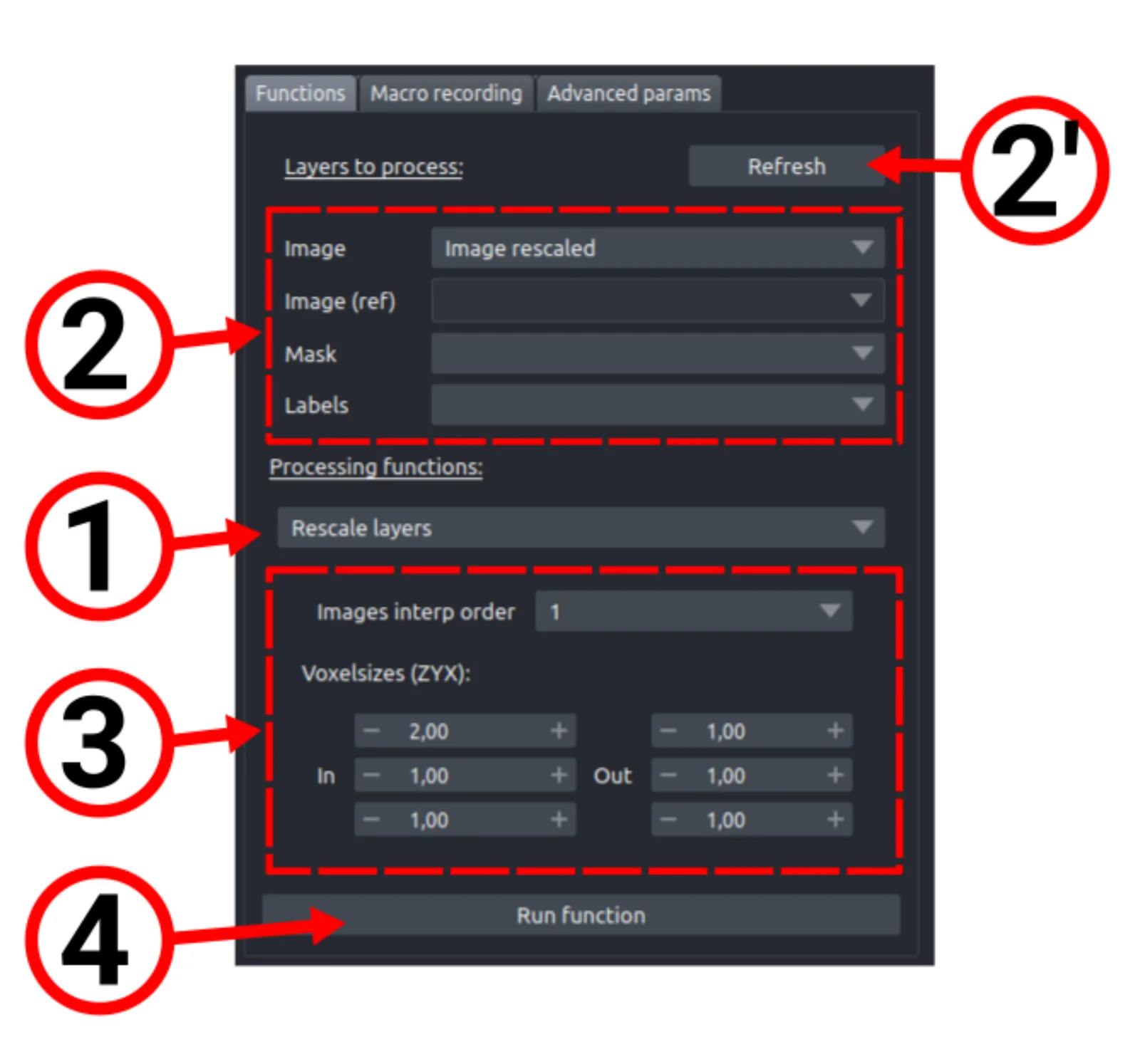
General step-by-step tutorial of napari-tapenade-processing. (1) Combo box to select the preprocessing function to apply. (2) Layer selection boxes for the function's inputs; a box is greyed out when the function needs no such layer. Image layers hold integer or float data, Labels layers hold integer data (segmented object instances), and Mask layers hold Boolean data (typically the sample's inside/outside mask); all layers must share the same shape and be 3D (ZYX) or 3D+time (TZYX). (2') If a layer is missing from a box but present in the Layer list, click *Refresh* to update it. (3) Function-specific parameters; click the [?] button next to a widget for a description. (4) *Run function* applies the function with the current parameters to the selected layers.

**2. Change layer voxel size**

**Input:** An image, a binary mask, or a segmentation.

**Output:** An image, binary mask, or a segmentation, resampled to a new array size such that the new pixel size fits the values provided.

Use this function if your image has a different resolution along Z than in XY and you need to make the dataset isotropic, for segmentation or visualization.

① Select your 3D array from the list.

② Enter your input voxel size (ZYX) in μm/pix and the desired output voxel size in the same format. For example, images from (13) have a voxel size of (1,0.25,0.25) μm/pixel, and we save them into the isotropic voxel size (0.5,0.5,0.5) to optimize nuclei segmentation.

③ Choose interpolation mode. Default mode is linear (interpolation order 1). If you process a Boolean array, for example, a binary mask, choose nearest (interpolation order 0) to keep a binary result. Cubic interpolation (order 2) means a smoother interpolated image, but takes longer to compute.

④ Select *Run function*. This creates a new layer, which is an image resampled to the desired pixel size.

*Note: Record the original and target voxel sizes in a .txt file or a metadata table. All downstream morphometric measurements depend on correct physical scaling.*

**Critical step:** If you aim to run the pipeline in its entirety, i.e., reach the physical analysis at the end, note that most processing steps require that the datasets are isotropic to have a correct physical interpretation.

**3. Compute mask from image**

**Input:** An image.

**Output:** A binary mask representing which pixels correspond to the interior of the biological tissue (value True, as opposed to exterior/noise, which have value False).

Use this function to create a binary mask separating the foreground (typically the tissue or organoid of interest) from the background voxels. It will be used in multiple steps later in the workflow (e.g., to remove spurious detected objects or to compute precise boundary-aware multiscale fields).

① Select your input image layer.

② Adjust sigma, which is the typical size of the Gaussian blur (Gaussian smoothing before thresholding). A typical value is ~ object radius / 3. Increase it to reduce noise; decrease it to preserve fine details.

③ Choose a thresholding method depending on your use case: *standard global thresholding* allows users to manually specify a threshold on the intensity value above which voxels are assigned to foreground, after Gaussian smoothing. This method is not robust to local intensity changes. The *otsu* method can be used to automatically find the best value of the global threshold, but suffers from the same weakness. The method *snp otsu* is slower but more robust to noise and intensity heterogeneities.

④ Adjust the Threshold factor: < 1 for a more permissive mask (larger object) and 1 for a stricter mask (smaller object).

⑤ Optionally select post-processing: *fill_holes* fills internal gaps (recommended for dense samples), and *convex_hull* computes the convex hull of the resulting mask. Enable *Keep largest connected component* to remove small spurious regions.

⑥ If your image was spatially registered and is thus padded with zeros, the thresholding step can wrongly assign background voxels to the foreground. Enabling *Registered image* can remove these background artifacts.

⑦ Optionally apply erosion (number of pixels) to shrink the mask by the given pixel amount (this also has the effect of smoothing the mask).

⑧ Click *Run function*. A new binary mask layer is created. Inspect this mask in both 2D and 3D to check whether it captures the structure of interest before using it in the next step.

**Critical step:** As the binary mask will be used on numerous occasions downstream in the pipeline, both for processing and for analysis, make sure to visually assess the correctness of the resulting mask. If possible, the mask should not overextend outside the desired “interior” volume by more than half the typical size of your objects of interest (e.g., nuclei, cells).

**4. Crop layers using mask**

**Input:** An image, binary mask, or a segmentation.

**Output:** An image, binary mask, or a segmentation, whose size has been reduced.

Use this step to reduce the data size by removing the empty background around the object, in case the biological object of interest is surrounded by a large amount of background pixels. Cropping reduces memory usage and speeds up processing.

① Provide the image, labels, or mask to crop, together with the mask that defines the bounding region.

② Optionally enable Add 1 pixel margin to avoid cutting too close to the object.

③ Click *Run* function. The code outputs cropped arrays restricted to the smallest box containing the mask.

**5. Apply local contrast enhancement**

**Input:** An image and, optionally, a binary mask obtained from the image.

**Output:** The input image, in which the local intensity histogram has been stretched to improve homogeneity. If a mask has been provided, intensity values outside the mask are not considered and are set to 0 in the result.

Use this step to improve image homogeneity and make structures easier to detect. These steps also rescale the histogram of the image in the [0,1] range. Select the input image to enhance and, optionally, the mask, if you want to restrict enhancement to the region of interest (recommended). Choose between:

1. Global enhancement (checkbox enabled): Rescales intensities using the intensity percentile value computed globally. Note that if your image suffers from intensity attenuation with depth, this approach will not enhance deeper regions to levels observed at the surface.

2. Local enhancement (checkbox disabled): Adapts contrast locally using sliding windows. This greatly improves the performance of CellPose-SAM and StarDist3D as demonstrated in [13].

① Set *Percentiles* (e.g., 1–99) to define intensity rescaling. Narrower ranges increase contrast but may saturate signals. If using local enhancement, adjust *Box size* (typical: ~1.5× object radius). Larger values produce smoother normalization; smaller values enhance local details.

② Click *Run function*; a new enhanced image layer is created.

**6. Segment the nuclei**

**Input:** An image; optionally, the locally enhanced image from the previous step (highly recommended).

**Output:** A segmentation (instance labels).

To segment 3D nuclei, you can use either StarDist3D (8) or CellPose-SAM [14].

1. StarDist3D: We originally used StarDist3D with a custom network trained on gastruloid (3D embryo-like stem cell aggregates) datasets acquired under multiple conditions; it is intended for approximately isotropic nuclei after resizing and intensity normalization. You can also use the pretrained models of StarDist3D.

2. CellPose-SAM. The advantage of CellPose-SAM is the possibility to predict a segmentation with the pretrained model, as well as the better precision of the prediction on non-star convex objects such as deformed nuclei.

We have shown in [13] that both algorithms benefited from the local contrast enhancement method developed in the processing notebook of Tapenade (section B5), so consider applying this preprocessing step before segmenting your data. The expected output is a 3D label image in which each nucleus has a unique integer identifier.

① Select the array to segment and the model folder where the weights are stored.

② Select the probability threshold: This is the threshold above which a pixel from the probability map is considered as being a center candidate. Lower values will result in more objects.

③ Select the NMS threshold: This controls how much overlap is permitted between detected objects during the *Non-Maximum Suppression* step. Higher threshold values discard more overlapping candidates that are considered redundant. The threshold should be high enough to suppress duplicate detections of the same object; setting it too high may also remove valid objects that are located very close to one another.

④ Click on *Run function* to obtain the prediction, which can take from a few minutes to a few hours, depending on the size of your image. If the segmentation is too long, consider one of these options:

i. Crop the image using its mask to remove background pixels.

ii. Downsample the image using the widget *Change array voxelsize* and decreasing the resolution.

**Warning**: If the function does not run on the plugin, check the Anaconda Prompt window for potential errors, such as wrong installation of Tensorflow or Pytorch.

**Critical step:** As the segmentation will be used at numerous occasions downstream in the pipeline, both for processing and for analysis, make sure to visually assess the correctness of the segmentation result.

**7. Remove labels outside of mask**

**Input:** A segmentation and a binary mask,

**Output:** The segmentation, in which instance labels partially or totally outside the mask are set to 0.

Use this function to potentially correct the segmentation predicted above, using the mask of your tissue.

① Select the mask and the labels (segmentation).

② Run the function. A new layer containing only the labels present in the binary mask will be displayed.

**8. Apply intensity normalization**

**Input:** A pair of images corresponding to two raw signals from the same biological tissue (one of them is a biological signal of interest and the other is a reference ubiquitous signal, i.e., a signal whose intensity is expected to be homogeneous in the biological tissue, provided there are no optical artifacts, e.g., Hoechst for nuclei) and, optionally, a binary mask and associated segmentation (highly recommended).

**Output:** An image corresponding to the biological signal of interest whose intensity values have been corrected for optical artifacts.

Use this function to normalize signal intensity across depth or between samples using a reference channel. This step is critical when comparing intensities across depth or between datasets. It is a different computational method from applying contrast enhancement:

1. *Local contrast enhancement* homogenizes the signal locally and is meant to be applied only before the segmentation step to improve accuracy.

2. *Intensity normalization* corrects volume-wide optical artifacts, including depth-dependent signal attenuation, while preserving biologically meaningful spatial patterns. The approach uses a reference signal, called ubiquitous, whose intensity is expected to be homogeneous throughout the sample in the absence of imaging artifacts (e.g., Hoechst staining). From this reference, an optical artifact field is estimated and then used to compensate for intensity loss in other channels. This removes intensity biases while preserving underlying gene expression patterns.

① Select the image to normalize and a reference image (e.g., structural or autofluorescence channel).

② Optionally select a mask to restrict normalization to the sample, and/or labels if normalization should account for segmented regions.

③ Set Sigma (scale of smoothing). If set to 0, it is estimated automatically.

④ Choose the image wavelength to account for optical attenuation differences: light penetration is wavelength-dependent, with smaller wavelengths having a stronger intensity decrease in depth. At the moment, the wavelength-dependent model is calibrated on the experimental setup presented in [13].

⑤ Adjust *Width of reference plane* if needed (default is typically sufficient).

⑥ Click *Run function*. A normalized image layer is created.

**Critical step:** As the normalized signals will be used directly downstream in the pipeline during analysis, make sure to visually assess the plausibility of the normalized results. For an additional sanity check, you can apply the signal normalization step on the input ubiquitous signal itself (effectively providing it twice). The resulting normalized ubiquitous array should show a homogeneous intensity distribution on all objects across the volume.

**9. Align layer from the mask’s major axis**

**Input:** An image, a binary mask, or a segmentation, and a binary mask of the same biological tissue.

**Output:** An image, a binary mask, or a segmentation, resampled to a new array such that the major axis computed from the input mask in the given plane is aligned to the axis provided.

Use this step to align the major axis of the object to a chosen axis. This is useful for standardizing orientation across samples.

① Provide the image.

② Choose the rotation plane (XY, XZ, YZ) where the major axis is computed.

③ Select the target axis to align the object with.

④ Choose interpolation order between 0 (nearest) for masks/labels, 1 (linear) standard for images, or 3 (cubic) smoother but slower.

⑤ Click *Run function*. A rotated, aligned layer is created.

**10. Apply masked Gaussian smoothing**

**Input:** An image and, optionally, a binary mask corresponding to the interior vs. exterior pixels from the same biological tissue. If the mask is provided, the user can also provide a second binary mask corresponding to finer details of where the signal from the input image really applies (i.e., a binary mask of nuclei only, or membranes only), which can be obtained by binarizing the result of the segmentation step. Providing both masks is highly recommended to allow for boundary-aware smoothing.

**Output:** An image corresponding to the input image whose features have been blurred at the given scale. If both masks are provided, only values inside the second mask are considered for local averaging, and all values outside the first mask are set to 0. If only the first mask is provided, the second mask is taken to be equal to the first.

Use this function to smooth a signal on large spatial scales while preserving boundaries defined by the mask. This can be used to visualize a coarse-grained gradient of genetic expression.

① Select an image and its mask.

② Adjust sigma, the spatial scale of smoothing.

③ Click *Run function*. A smoothed image is created.

**D1. Run processing in batch**

To run functions in batch, you can use either the macro tab from napari-tapenade-processing or the Jupyter Notebook preprocessing_notebook.ipynb. This section presents:

1. How to record a macro.

2. How to run a previously recorded macro.

**1. Record macros**

To record a macro, follow the instructions below (see Figure 8).

① Click on *Choose directory* to select a folder where the macro file will be saved.

② Click on *Start recording macro* to start recording the functions you will apply. At this point, you can start applying sequences of functions to images/segmentations/masks that you have already loaded in Napari or that you load in the middle of the recording.

③ When you are finished, click *Stop recording and save macro*. It will be saved in the JSON (.json) format, and the name will follow the pattern recorder_parameters_YYYY-MM-DD_HH-MM-SS.json.

**Figure 8.**
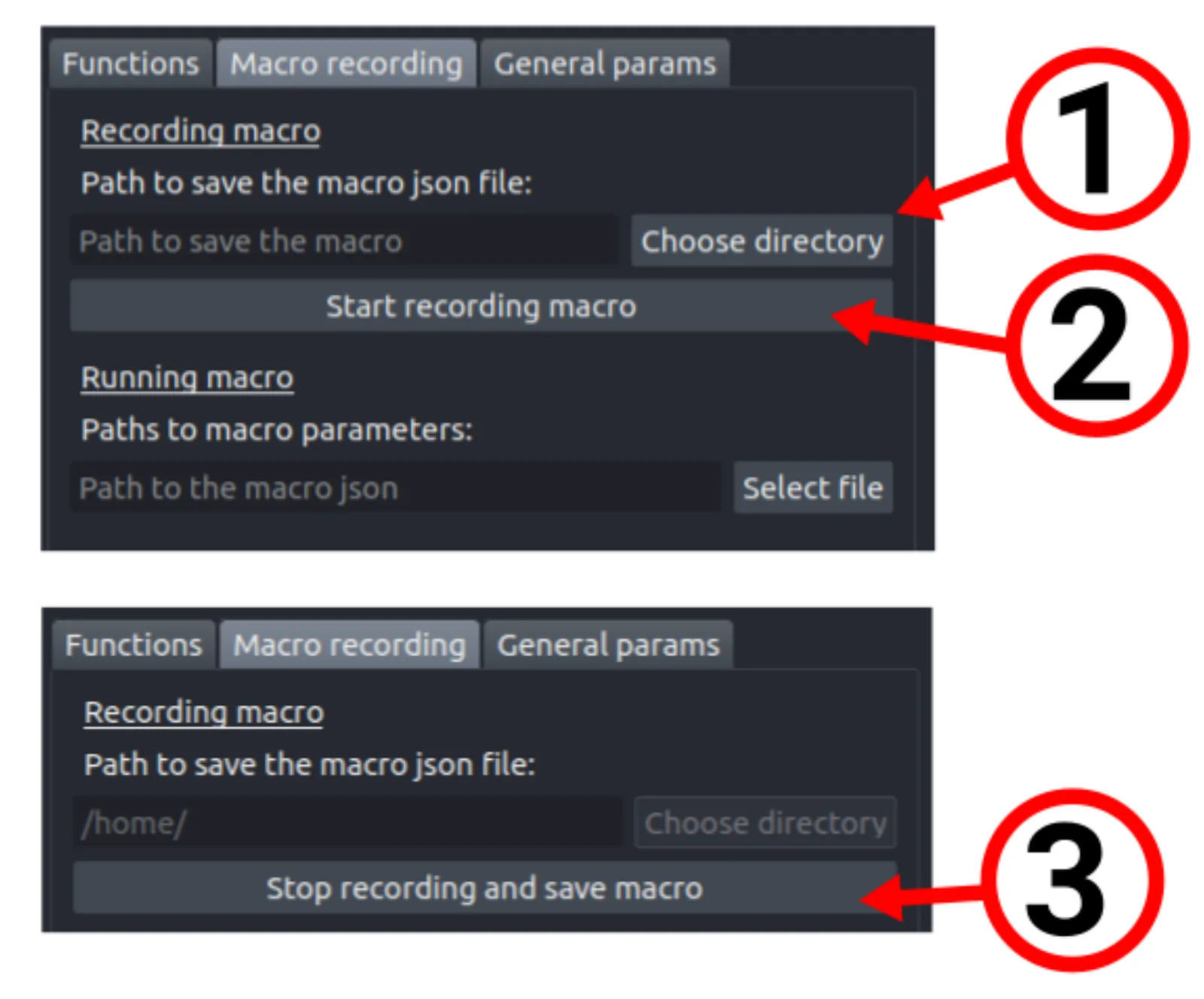
Step-by-step tutorial to record a macro in napari-tapenade-processing. (1) Click *Choose directory* to set where the macro file will be saved. (2) Click *Start recording macro*, then apply your sequence of functions to layers already loaded or loaded during recording. (3) Click *Stop recording* and save macro.

**2. Running macros**

To run a macro, follow the instructions below (see Figure 9). Macros allow you to run a sequence of functions in batch on folders of TIFF images (e.g., different frames of the same 3D + t image, or several channels of a 3D image). The input images should be in the same folder, and the output will be saved in a folder of your choice. The output of each function will be saved in separate subfolders for each step of the pipeline. To run a macro, click on the *Macro recording* tab and follow these steps:

① Click on *Select file* to choose the macro file you want to run.

② After specifying the path to the macro file, several path entries with names like “Path to folder ([...]) N” (e.g., “Path to folder ([‘Image’] 1”) will appear.

③ Click on *Choose directory* under *Path to save output folders of tifs* to select the folder where the results of the pipeline will be saved.

④ You can click the *Compress when saving* checkbox to save the output TIFF images in a compressed format using ZLIB compression.

⑤ Choose the number of workers to use for parallel processing. The default value is 1, which means that the functions will be run sequentially on the images. If you have a multi-core CPU, you can increase this value to speed up the processing. Be careful that setting this value too high can lead to memory issues.

⑥ Click on *Run macro* to start the processing. Once your image is processed, use the analysis modules of the pipeline to compute and correlate spatial features.

**Figure 9.**
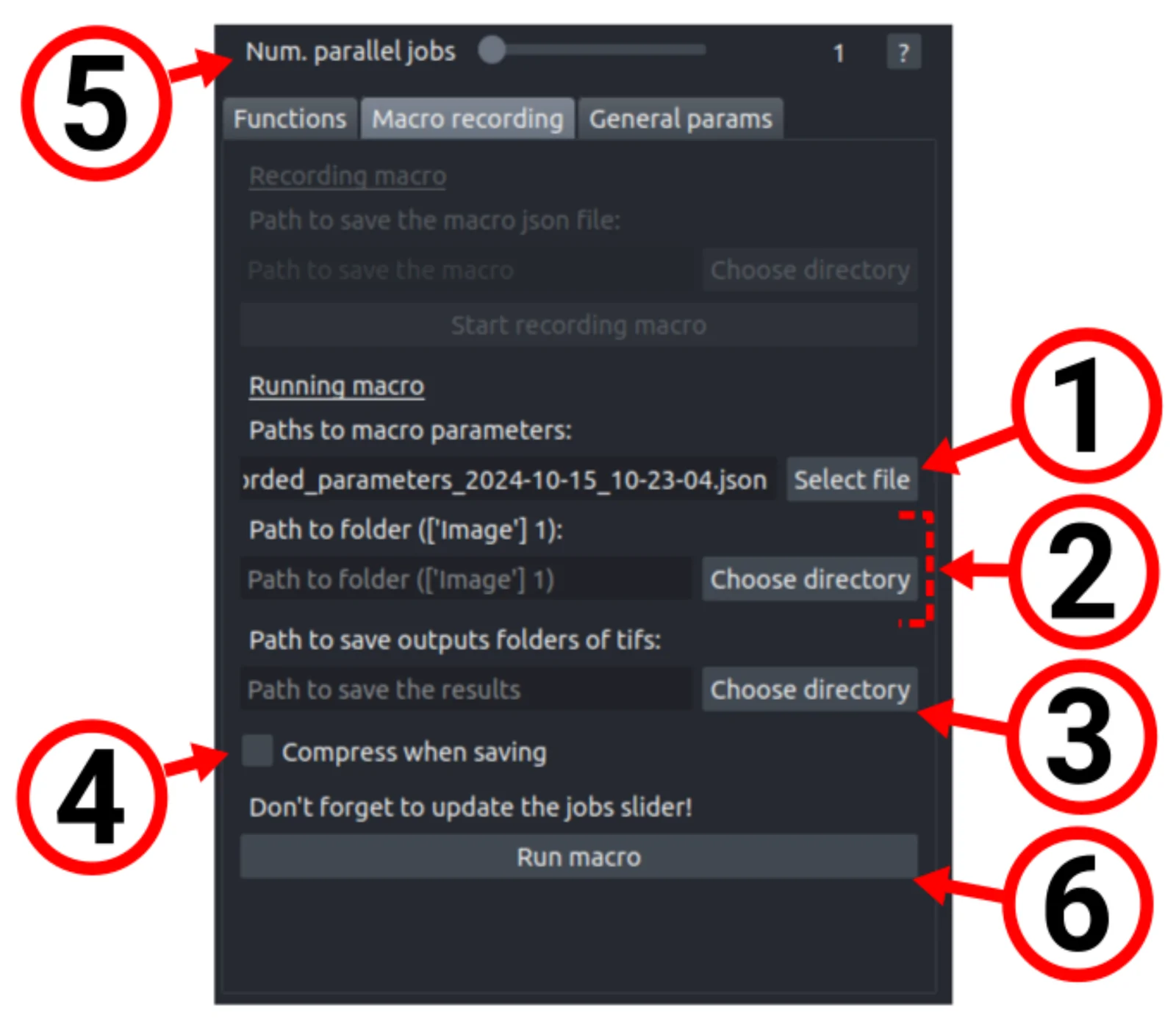
Step-by-step tutorial to run a macro in napari-tapenade-processing. (1) Click *Select file* to choose the macro file to run. (2) Once the path is set, entries named "Path to folder ([...]) N" (e.g., "Path to folder (['Image']) 1") appear for the input folders. (3) Under *Path to save output folders of tifs*, click *Choose directory* to set where results are saved. (4) Optionally tick *Compress when saving* to write the output TIFFs with ZLIB compression. (5) Set the number of workers for parallel processing; the default 1 runs sequentially, and higher values speed up processing on multi-core CPUs but risk memory issues if set too high. (6) Click *Run macro* to start, then use the pipeline's analysis modules to compute and correlate spatial features.

## Analysis module

This module requires processed data, a segmentation of the object of interest (nuclei, cell, ...), and a binary mask of the tissue of interest.

One of the features of the pipeline is to allow probing multiscale measurements. To probe volumetric signals at different length scales *σ* ranging from the average nuclei size (cellular scale) to the width of the organoid (coarse-grained scale), we convolve signals with a Gaussian kernel of variable size *σ* while precisely accounting for domain boundaries and signal expression domains. This scale at which we probe the tissue can be set in the different notebooks and plugins. Examples of variations of this scale can be found in [13], and the implementation of the method is detailed in the notebook masked_gaussian_smoothing_notebook.ipynb.

**E. Spatial correlation analysis**

**Input:** One or several normalized (optical-artifacts have been corrected) images corresponding to biological signals of interest, a segmentation of the objects of interest (nuclei, cell, ...), and a binary mask of the tissue of interest.

**Output:** A plot showing the 2D spatial correlation histogram of two quantities (e.g., two biological signals, or one signal and the field of object density as obtained using the segmentation) at a given spatial scale.

This plugin allows the user to analyze the spatial correlations of two 3D fields loaded in Napari (e.g., two fluorescent markers). Use this plugin to compare two 3D fields, for example, cell density vs. marker intensity, or the expression of two different genes, or two morphometrics fields, such as nuclear volume vs. nuclear deformation amplitude.

*Note: If you quantify gene expression gradients, the images should not display any intensity artifacts, for example, due to light absorption in depth. Normalize the intensity using the function from the processing module (see section D), with a ubiquitous marker to correct these artifacts. Otherwise, correlation maps will be biased by intensity artifacts; see demonstration in [13].*

The Jupyter notebook spatial_correlation_analysis_notebook.ipynb includes the same functions as the plugin to plot correlation maps. To use the plugin, follow the instructions below (see Figure 10).

**Figure 10.**
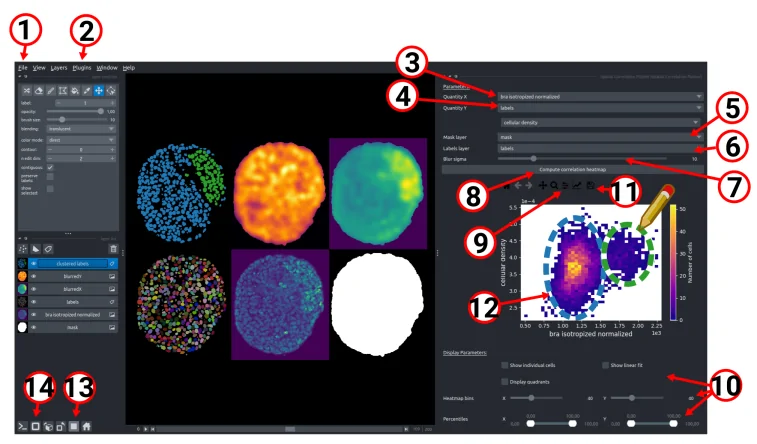
Step-by-step tutorial on napari-spatial-correlation-plotter. (1) Load your images (and optionally mask and labels) into Napari by drag-and-drop or *File* > *Open files*. (2) Open the plugin via *Plugins* > *Spatial Correlation Plotter*. (3) Select the first layer in the *Quantity X* combo box. (4) Select the second layer in *Quantity Y*. A *Labels layer* can be used as either quantity to study object-instance density (here, nuclei density, equivalently cell density) or instance volume fraction. (5) Optionally select a mask to restrict the analysis to a region. (6) Optionally select a label layer so the histogram is binned on values averaged over segmented instances (e.g., one point per nucleus); otherwise, individual voxel values are used. (7) Use the Blur sigma slider to set the analysis length scale (standard deviation of the Gaussian kernel for masked smoothing of the 3D fields); 0 applies no smoothing, useful for raw or already-smoothed data. (8) Click *Compute correlation heatmap* to plot it. (9) If the plot is cut off, use *Configure subplots* > *Tight layout* to adjust its size. (10) Tune binning with the *Heatmap bins sliders* and the *Percentiles sliders* to exclude outliers. Optional overlays are *Show individual cells* (scatter of the underlying points), *Show linear fit*, and *Display quadrants* (vertical and horizontal lines at the median X and Y values), as in Figure 11. (11) Click *Save the figure* to export the plot. (12) Interact with the heatmap by drawing a region of interest directly on it to select the corresponding cells in 3D on an independent clustered labels layer: left-click for a freehand shape, right-click for a rectangle, and hold Shift to build several colored groups (13) Click *Toggle grid mode* to display all layers in a grid (right-click to set rows/columns). (14) Click *Toggle 2D/3D view* to switch views.

① Load your images (and, optionally, mask and labels) in Napari. You can drag and drop them from your file explorer to the Napari viewer or open them using the *File > Open files...* menu.

② Click on the *Plugins > Spatial Correlation Plotter* menu to open the plugin.

③ Select the first layer you want to study from the combo box *Quantity X*.

④ Select the second layer you want to study from the combo box *Quantity Y*. In this example, labels have been loaded in step 1. Label layers can be chosen as *Quantity X* or *Quantity Y* so that the quantity to study is the object instance density (in this example, the labels come from nuclei segmentation, so this leads to studying the nuclei density, or equivalently, the cell density) or instance volume fraction.

⑤ Optionally, a mask can be selected to restrict the analysis to a specific region of the image.

⑥ Optionally, a label layer can be selected so that the histogram is constructed by binning values obtained by averaging the two fields in the segmented instances. For example, if a layer of segmented nuclei is additionally specified, each point corresponds to an individual nucleus. Otherwise, individual voxel values are used.

⑦ Use the *Blur sigma* slider to vary the length scale of the analysis. This corresponds to the standard deviation of the Gaussian kernel used for masked Gaussian smoothing of the 3D fields. If set to 0, no smoothing is applied, which can be useful to study the raw data or if the quantities are already coming from smoothed data.

⑧ Click on *Compute correlation heatmap* to compute and plot the correlation heatmap.

⑨ If the image does not properly fit in the window (e.g., if the borders are cut), you can click *Configure subplots > Tight layout* to adjust the plot size.

⑩ You can adjust the histogram binning by changing the *Heatmap bins* sliders. If the histogram range is too large (e.g., due to outliers), you can adjust the *Percentiles* sliders to focus on the most relevant part of the histogram. You can also check options to:

a. *Show individual cells* to display the individual points that compose the histogram as an additional scatter.

b. *Show linear fit* to display the linear fit of the histogram.

c. *Display quadrants* to display vertical and horizontal lines that divide the histogram into four quadrants. The lines are placed at the median of the histogram values in X and Y. For instance, once checked, these options lead to the plot shown in Figure 11.

**Figure 11.**
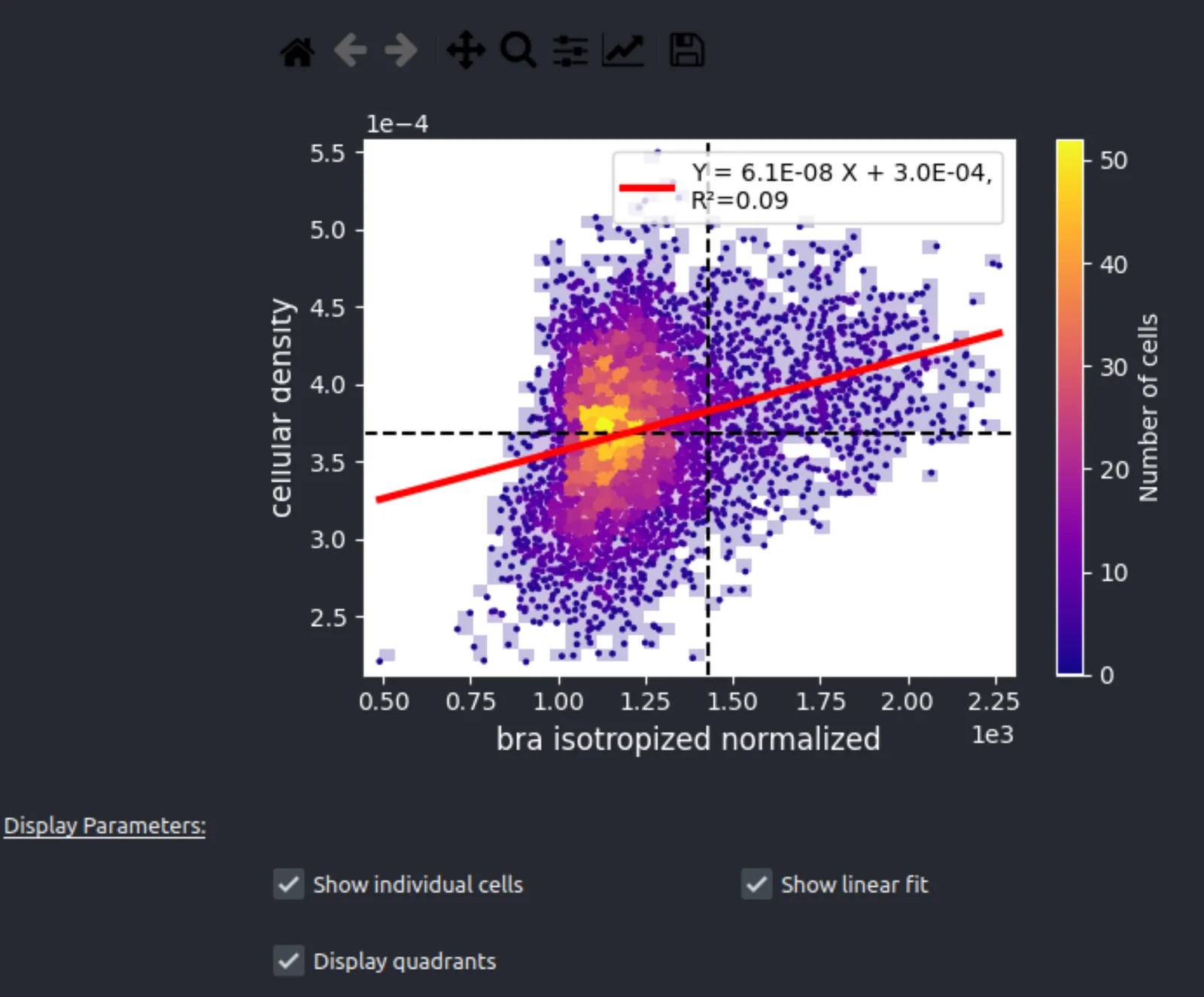
Example of correlation map displayed in napari-spatial-correlation-plotter

⑪ You can click on *Save the figure* to save the current plot (many formats are available, including .png, .svg, and .jpeg).

⑫ You can dynamically interact with the correlation heatmap, manually drawing a region of interest directly on the plot to automatically select and display the corresponding cells in 3D on an independent Napari Label layer, *clustered labels*. By left-clicking, you can draw an arbitrary shape. By right-clicking, you can draw a rectangle. If *Shift* is pressed while drawing, several groups of cells can appear on the *clustered labels* layer, each with a different color. This was inspired by the plugin napari-clusters-plotter [15].

⑬ To trigger the grid view like in the example image, click on *Toggle grid mode*. This will display all layers in a grid view. By right-clicking the button, you can parametrize the grid view (e.g., number of columns, number of rows).

⑭ Switch between 2D and 3D view by clicking on *Toggle 2D/3D view.*

Interpretation: Each point corresponds to a single nucleus if a segmentation was provided. The heatmap reads as a histogram, so yellow points indicate that many cells are located in this region of the graph. The two correlated fields need not both be genetic: one field can be a morphometric map, such as the cell density map (Figure 12e). Correlating cell density with gene expression shows, from a linear fit, that cells expressing a given gene have a higher local cell density. As a gene-gene example, see Figure 13c for an example of a correlation plot between the genes *FoxA2* and *Oct4*, marking, respectively, endoderm formation and pluripotency. The top-right quadrant is almost empty, meaning very few cells express both high *FoxA2* and *Oct4*. Many cells fall in the bottom-right and top-left quadrants (high *FoxA2*/low *Oct4* and low *FoxA2*/high *Oct4*), showing that the two markers are mutually exclusive. One can also correlate a morphological feature (such as cell density) with gene expression and conclude, from a linear fit, that cells expressing a given gene have a higher local cell density, for example. Using the plugin, one can interactively map the cells of specific clusters onto the microscopy image to understand where each population is located.

**F. Morphometric measurements**

**Input:** A segmentation of the objects of interest (nuclei, cell, ...) and a binary mask of the tissue of interest.

**Output:** 3D visualization of morphometric fields (e.g., cell density, volume fraction, nematic order) at a given spatial scale. Optionally, these fields can be saved, e.g., as TIFF files from the Napari window or from the notebooks for downstream uses.

Run the Jupyter notebook spatial_features_morphometrics_notebook.ipynb (see step B5 to find the notebooks) to compute more advanced spatial features in 3D.

In this section, subsections indexed via roman numerals [e.g., (i), (ii), …] indicate the content of the explanatory text cells from the notebook.

① Load the data and choose parameters

(i) Load your own dataset: processed image data, the nuclei labels, and the tissue mask. We advise using an isotropic voxel size to obtain measurements in physical units, such as (1,1,1) μm/pix, for example.

(ii) Tune the smoothing parameter sigma, the size of the Gaussian smoothing. A typical value is ~ object radius / 3. Increase it to reduce noise, decrease it to preserve fine details.

(iii) Sanity check: Before computing any feature, adjust the labels using the function clear_borders if needed, to remove labels touching the image border when their shape is not reliable. The code also ensures beforehand that the image, the labels, and the mask have the same shape.

(iv) Visualization of the raw data used to compute spatial features, using Napari.

② Compute the field of gene expression

Run this step if you want to compute a spatial coarse-grained map of gene expression across the sample. The images should not present any intensity artifacts, for example, due to light absorption in depth. If necessary, use the function normalize_intensity from notebook processing_notebook.ipynb or the Napari plugin using a ubiquitous marker to correct for these artifacts.

(i) Perform sanity checks on image dimensions and checks for NaN (“Not a Number”) values, which would create errors in the following functions.

(ii) The function process_genetic_field_cellular computes the average genetic signal for each nucleus. The function smooth_cellular_map applies a Gaussian blur of size sigma to obtain a coarse-grained distribution of genetic field intensity in the tissue.

(iii) 3D visualization of the map of gene expression in Napari.

③ Compute a map of spatial position in the tissue

Use this step to compute coarse-grained positional maps, such as radial or axial positions. Use these maps in napari-spatial-correlation-plotter to correlate spatial position with other quantities (e.g., to show the spatial pattern of a genetic marker).

(i) The code assigns to each nucleus its distance to the borders (radial map) or its position along the XY axis (axial map; use the axis alignment function of napari-tapenade-processing if the largest axis of the object is not aligned onto XY). Then, the maps are smoothed using a Gaussian blur of size sigma.

(ii) 3D visualization in Napari.

④ Compute cell density and density gradients

Interpretation: The cell density map shows local packing. Its gradient highlights the sharp boundaries and transition zones between loosely and densely packed regions, marking sites of tissue reorganization (see Figure 12e for an example).

For visualization purposes, some features are computed at regular values on a grid instead of densely on all pixels to reduce the amount of plotted information.

(i) Tune the grid spacing in x, y, and z. A regular grid is created from these parameters.

(ii) A colormap function for orientations is defined for future vectors. The function process_cell_density_sigma computes a scalar field of object density, and the function process_density_gradient computes the gradient of this scalar field. To visualize the density gradient, we normalize and project it onto the XY plane.

(iii) 3D visualization of the scalar fields (density, magnitude of the density gradient).

(iv) 3D visualization of the vector fields (the orientation field of the density gradient and its projection).

⑤ Compute volume fraction and object volume

Interpretation: An increase in cell density can come from a higher object volume or a higher compaction. We thus quantify volume fraction, i.e., how much space is occupied by the objects (nuclei or cells), and object volume across the sample. These two features are complementary to cell density to understand differences in tissue organization.

(i) The code computes a coarse-grained map of local space occupied by objects in a neighborhood of size sigma. It also computes a map of object volume and smoothes it with a Gaussian blur.

(ii) 3D visualization of the maps of volume fraction and object volume.

⑥ Compute nematic order

Interpretation: The nematic order characterizes the local alignment of orientations of the segmented objects, for example, the nuclei. It is a scalar value between 0 (random orientations) and 1 (all objects are aligned in the same orientation). It does not quantify the deformation itself, as elongated objects in different orientations (for example, around a lumen) will have a low nematic order. It rather reflects how spatially consistent the nuclei orientations are in a local neighborhood.

(i) The code computes a coarse-grained map of local orientational order. A field of tensor orientation is computed at the cellular scale, for each object, from its main axis. The orientation field is then smoothed with a Gaussian blur. We extract the nematic order from the largest eigenvalue of the nematic tensor.

(ii) 3D visualization of the nematic order.

⑦ Compute the nuclei ellipsoidal coefficient

Interpretation: Ellipsoidal objects under external mechanical forces exhibit two modes of deformation: prolate and oblate. Prolate deformation, characterized by elongation along the longest axis (such as a rugby ball), arises from anisotropic tensile stresses. Oblate deformation, characterized by flattening perpendicular to the shortest axis (such as the shape of the Earth), reflects compressive or shear stresses. Quantifying prolate and oblate scores on deformable nuclei thus reveals whether tensile or compressive forces dominate in the tissue and how nuclei react to mechanical cues in their micro-environment [16].

(i) We introduce two methods for characterizing the ellipsoidal coefficients of nuclei:

- Distinct scores for oblate and prolate.

- A unique score that is negative if oblate, positive if prolate, and 0 if the object is spherical or both oblate and prolate.

(ii) The code computes minima and maxima for better visualization and displays the 3D maps on Napari.

⑧ Compute nuclei elongation

Interpretation: Nuclei deform under mechanical stress and can indicate directions of morphogenetic flows. Nuclei elongation is computed from the maximum eigenvalue of the true strain tensor for each nucleus.

(i) The code computes the true strain tensor of each nucleus. Its maximum eigenvalue reflects object elongation. This cellular map is then smoothed with a Gaussian blur of size sigma.

(ii) 3D visualization of the cellular-scale field (one scalar per object, here, nuclei) and the coarse-grained field (smooth cellular map).

*Notes:*

*1. Each feature is displayed in an independent Napari viewer by default, although they can be combined into one viewer by removing or commenting the following lines:*

*viewer = napari.Viewer()*

*and*

*napari.run()*

2. *Contrast limits can be adjusted during visualization if needed, using the sliders in Napari. They can also be set in the notebook to keep the same min and max values for different samples.*

*viewer = napari.Viewer()*

*min = …*

*max = …*

*viewer.add_image(feature,constrast_limits=[min,max])*

*napari.run()*

**G. Deformation fields**

Section F presented scalar features to quantify how much nuclei are elongated, whether forces are tensile or compressive, and how consistently they are oriented (nematic order). In this section, deformation fields capture both the magnitude and the direction of local deformation as spatially averaged tensor fields. By combining inertia tensors (object shape) and true strain tensors (pure deformation) across scales, this analysis reveals the anisotropy and directional patterns of tissue reorganization.

Use the notebook deformation_analysis_notebook.ipynb (see section B5 to find the notebooks) when object shapes are sufficiently well segmented and you want to quantify local anisotropy or deformation patterns. It focuses on multiscale analysis of deformation fields using inertia tensors and true strain tensors.

We use the skimage.measure.regionprops function to compute the centroid, inertia tensor, and true strain tensor of each segmented object.

① Load the example dataset or provide your own image, its pixelsize, the labels of each object (here, the nuclei), and optionally, a mask of the sample. If you do not have a mask or labels, see section D to generate them.

② The code builds sparse arrays, i.e., arrays of dimension (n_points, n_dim space + n_dim points), where n_dim space is the number of spatial dimensions (2 or 3), and n_dim points is the number of dimensions of the signal at each point, e.g., 1 for a scalar field, 3 for a 3D vector field, 9 for a 3 × 3 matrix field, etc.

We then extract the inertia tensor and the true strain tensor for each segmented object, giving geometric and mechanical descriptors.

③ Average the tensors locally.

(i) Specify a scale of analysis *sigma*, which corresponds to the standard deviation of the Gaussian kernel used to average the inertia tensor and the true strain tensor. Smooth the sparse tensors to obtain the average tensors, defined at each centroid.

(ii) For clarity, tensors can also be represented on a regular grid. The code illustrates how to compute the averaged tensors on a regular grid of step 20 pixels; tune it later if necessary.

(iii) We recompute new average volumes at the grid positions.

④ Quantify deformation and alignment. The code extracts the objects’ principal length from the inertia tensors and the main eigenvalue of the true strain tensors by diagonalizing the tensors. Vectors are formatted in an array V shape (n_objects, 2, dim_vector), where V[i,0] corresponds to the position at which the vector *i* is defined, and V[i,1] corresponds to the vector itself.

The angles returned are the angles between the main eigenvector and the X axis. We apply the same method for our tensors computed on the regular grid.

⑤ Visualize the resulting vectors together with density gradients or marker gradients to identify aligned or anti-aligned regions. The notebook converts tensors into vector representations that can be visualized. The output is an interactive visualization of scalar maps at the cell level (e.g., deformation magnitude) and vector tensor fields (e.g., principal directions) after spatial averaging.

Interpretation: We plotted the major axis of the true strain tensor of the nuclei on Figure 12e, which shows consistent nuclei deformation and alignment around the gastruloid pole. This deformation is correlated with the low-to-high density boundary, meaning that the nuclei were elongated along the boundary, i.e., perpendicular to the density gradient. This is consistent with a boundary where mechanical stresses build up between the anterior and posterior poles.

*Notes:*

*1. Inertia tensors are sensitive to object size and may bias averages; true strain tensors are more appropriate for comparing pure deformations across objects.*

*2. Interpret deformation fields only after confirming that segmentation quality is stable across depth. Strong anisotropy or systematic under-segmentation along z can bias tensor-based measurements.*

## Validation of protocol

This protocol has been used and validated in the following research article:

- Gros et al. [13]. A quantitative pipeline for whole-mount deep imaging and analysis of multi-layered organoids across scales. *eLife* (Figures 4, 5, S2, S6, S8, S9, and S10)

The workflow was validated on images of 3D embryonic organoids called gastruloids, which self-organize from mouse embryonic stem cells. Gastruloids develop similarly to mouse embryos during and after gastrulation. We established an experimental and computational pipeline to understand the coordination between tissue architecture and cell fate during gastruloid morphogenesis across multiple spatial scales. The experimental module of the pipeline consists of imaging immunostained gastruloids in toto using multicolor two-photon microscopy. Given high developmental variability, we used multiposition imaging to be able to analyze samples in high throughput. We developed the computational workflow of Tapenade to process 3D images automatically and analyze tissue physical properties in correlation with gene expression patterns.

Gastruloids are highly packed tissues that scatter light and exhibit heterogeneous nuclei shapes and dynamics. This limits deep imaging and possibilities for cell-scale quantification of tissue properties. To achieve *in toto* imaging, we used a dual-view procedure and developed a registration module to register and fuse both views. This module produced coherent 3D reconstructions of large samples (100–500 μm) and preserved cellular resolution across the sample (Figure 12a). We used two open-source state-of-the-art methods for 3D nuclei segmentation, CellPose-SAM and StarDist3D, to segment whole-mount gastruloids after registration. Using manual annotation, we showed that our custom StarDist3D model obtained good performances in 3D, with an F1 score of 85%. The Tapenade pipeline improved both StarDist3D and Cellpose-SAM outputs by using local contrast enhancement to increase signal in depth (z > 150 μm) (Figure 12b). We found that CellPose-SAM was highly competent to segment elongated nuclei in gastruloids (Figure 12c) even without re-training or fine-tuning.

**Figure 12.**
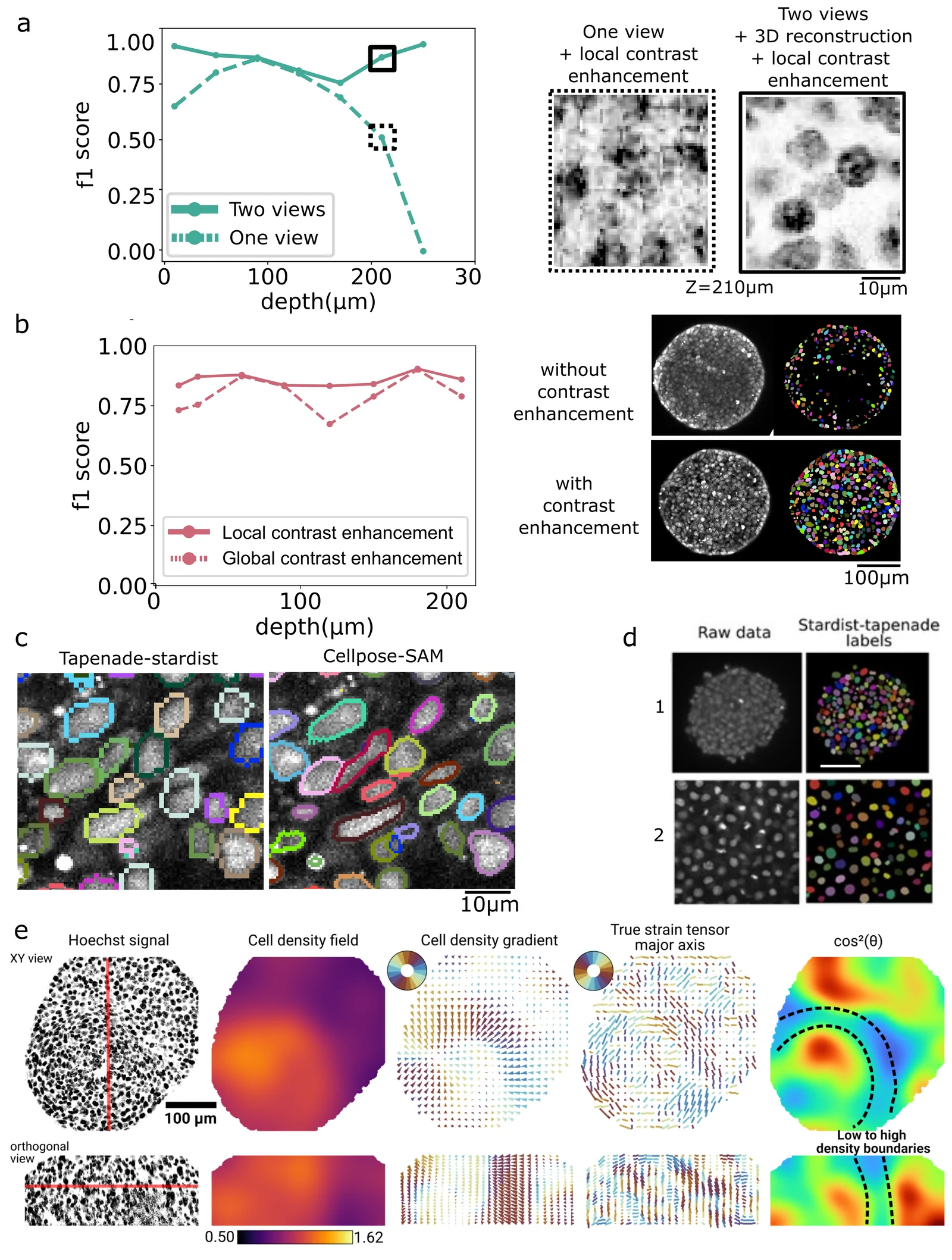
Validation of nuclei shapes analysis. (a) Dual-view imaging and registration, as well as local contrast enhancement, allow us to recover nuclei in toto in a 300 μm gastruloid. Left: F1 score of the 3D nuclei segmentation performed by our custom StarDist3D network with one view and with dual-view, registration, and fusion. Right: corresponding images at 210 μm depth. (b) Our local contrast enhancement method improves the signal in deep regions and thus the segmentation quality for both StarDist3D and CellPose-SAM. Left: F1 score of the 3D nuclei segmentation performed by our custom StarDist3D network with local contrast enhancement and with global contrast enhancement. Right: Images at the midplane and their corresponding nuclei segmentations performed by CellPose-SAM. (c) Compared to StarDist3D, CellPose-SAM performed better in regions with elongated nuclei. (d) Our custom StarDist3D approach performs qualitatively well on diverse datasets such as (1) gastruloid stained with the nuclear marker DRAQ5 imaged with an open-top dual-view and dual-illumination LSM [18]; (2) fixed zebrafish embryo stained for nuclei and imaged with a Zeiss LSM 880 confocal microscopy [19]. (e) Using the morphometrics module of the pipeline, we show the presence of a sharp boundary of cell density gradient spatially colocalized with high nuclei alignment. We further quantify this observation by showing the quantity cos^2^ (*θ*), with *θ* being the angle between the gradient of cell density and the major axis of the true strain tensor. Note that the expected value of this metric for random orientations is 1/3, and the value approaches 0 or 1, respectively, for perpendicular and parallel orientations. The upper row shows XY views, and the lower row shows orthogonal views.

It also qualitatively matched our custom StarDist3D model on metrics related to cell packing (e.g., fields of local density or nuclear volume) or deformation (e.g., true-strain), while also showing consistent results with no depth-dependence. Moreover, we showed that our StarDist3D model generalized well to other datasets (Figure 12d).

Validation of the nuclei shapes analysis was performed by assessing the biological consistency and reproducibility of the tissue properties. From nuclei segmentations, the pipeline accurately reconstructed 3D fields of cell density, nuclear volume, and deformation, revealing spatially heterogeneous tissue-scale patterns. Morphometric analyses based on nuclear shape and deformation tensors reproduced expected mechanical signatures, such as aligned nuclear elongation along tissue boundaries (Figure 12e), consistent with the interpretation of this boundary between anterior and posterior poles as a region of high stress. Cell and nuclear shape and deformation are established readouts of the local mechanical state of a tissue (17), but it remains rarely visualized directly in 3D tissues.

A key issue when analyzing gene expression patterns in deep tissues is the gradient of signal intensity optically induced by light penetration. We developed a correction method based on local nuclei intensity to normalize the channels of interest and extract unbiased values of gene expression. This method was validated across gastruloids from different acquisitions and imaging conditions (see Figure 13a). We developed a parameter-free algorithm that optimizes the value of the Gaussian kernel to lead to the most homogeneous signal, as well as a wavelength-dependent model. In our study, this normalization scheme was used to analyze gene expression gradients, such as the pattern of T-Bra, a mesoderm marker, during gastruloid development. The wave pattern of T-Bra observed after processing (Figure 13b) is consistent with previous observations [20].

**Figure 13.**
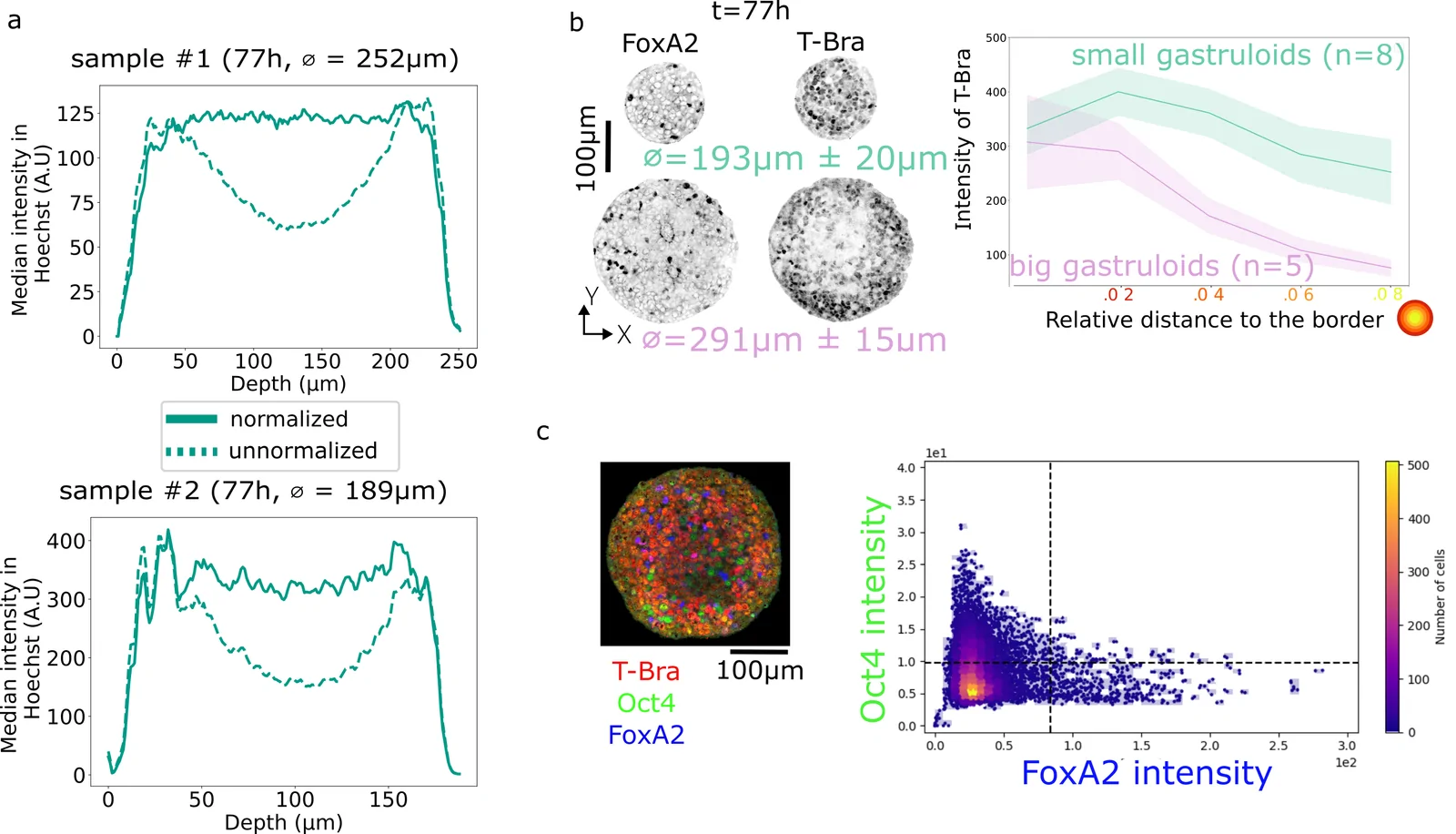
Validation of the gene expression analysis. (a) Depth profile of the median intensity of Hoechst (A.U.) on two gastruloids. Before normalization, the middle of the dual-view profile reaches a minimum of intensity due to light absorption. After normalization, the profile is corrected and stays constant in depth. (b) After applying the normalization scheme to other channels, we explore patterns of gene expression at the coarse-grained scale with the radial profile of T-Bra expression for gastruloids of different sizes, and (c) at the cellular scale, with the correlation heatmap between FOXA2 (endoderm) and T-Bra (mesoderm) expression.

Using the pipeline, we were then able to reliably quantify multiple expression markers across samples, stage, and size. Coarse-grained analyses, such as radial profiling and spatial averaging, produced reproducible expression patterns, while single-cell correlation analyses allowed us to explore the correlation of specific markers within each nucleus. For example, FOXA2 (a marker for endoderm expression), also expressed in the tissue periphery like T-Bra, actually showed anti-correlation with T-Bra at the cellular scale (Figure 13c), demonstrating the importance of multiscale analysis to explore germ layer specification. This was only possible using the combination of accurate processing to remove optical artifacts and 3D visualization tools such as Napari plugins to explore spatial patterns interactively.

Finally, the same interactive tools supported qualitative validation of the pipeline parameters by enabling direct inspection of intermediate outputs at each step. This ensured that processing, segmentation, and analysis results remained interpretable and consistent.

Together, these results demonstrate that the Tapenade computational workflow provides accurate, robust, and scalable processing of dense 3D imaging datasets, enabling reliable extraction of quantitative features from the cellular to tissue scale across diverse experimental conditions.

## General notes and troubleshooting

**Troubleshooting**

**Problem 1:** When opening a multichannel image in Napari, clicking *Toggle 3D view* does not actually show the object in 3D, and instead shows channels as the third dimension, with the slider being attached to depth.

Possible cause: The image has incorrect dimension ordering, so the Z (depth) and channels dimensions are swapped.

Solution: To change the dimension ordering of your 3D image, use the plugin napari-tapenade-processing and its widget *Reorganize Array Dimension*. Check if the dimensions correspond to their size. If not, update the ordering and run the function (do not click *Keep original file*). Now, clicking *Toggle 3D view* should display a 3D visualization with the bottom slider changing the channel.

**Problem 2:** When managing multiple layers in Napari (e.g., when opening several images), some sliders become inactive, and navigating the data becomes confusing.

Possible cause: The layers opened in the Napari window contain data with different shapes (for example, a 3D image and a 4D image) or different shape formats (for example, if you opened an .nd2 and a .tif at the same time).

Solution: Images opened in the same Napari window should have the same shape format and the same number of dimensions (two 3D images, for example). To visualize the shape of your image, use the plugin napari-tapenade-processing and its widget *Reorganize Array Dimension*. To open a separate Napari window for another set of images, you can open another terminal (e.g., on Windows, right-click the Anaconda Prompt icon in the taskbar and click Anaconda Prompt again). From there, open your environment and run Napari:

conda activate env-tapenade

napari

**Problem 3:** Running a function freezes the Napari window.

Possible cause: The window freezes while functions are running. Running computationally heavy functions like segmentation can result in the window staying frozen for several minutes.

Solution: Let the function fully run until the window is responsive again. If you cannot let the function run until completion, you can close the Napari window while the function is running (if it does not directly work, close the terminal you used to open the Napari window).

**Problem 4:** The bottom of the Napari window (e.g., sliders) is no longer visible after opening the napari-tapenade-processing plugin.

Possible cause: On small screens or low resolutions, the Napari window is sometimes resized beyond the visible screen area.

Solution: Either increase your screen resolution or undock the plugin from the Napari window by clicking the square button *float this panel* at the top-left of the plugin column.

## References

[1] Lancaster M. A. and Knoblich J. A. (2014). Organogenesis in a dish: Modeling development and disease using organoid technologies. Science. 345(6194): e1247125. https://doi.org/10.1126/science.1247125 25035496

[2] Dekkers J. F., Alieva M., Wellens L. M., Ariese H. C. R., Jamieson P. R., Vonk A. M., Amatngalim G. D., Hu H., Oost K. C., Snippert H. J. G., .(2019). High-resolution 3D imaging of fixed and cleared organoids. Nat Protoc. 14(6): 1756 1771 1771. 10.1038/s41596-019-0160-8 31053799

[3] Gómez-Gaviro M. V., Sanderson D., Ripoll J. and Desco M. (2020). Biomedical Applications of Tissue Clearing and Three-Dimensional Imaging in Health and Disease. iScience. 23(8): 101432 10.1016/j.isci .2020.101432 PMC7452225 32805648

[4] Stooke-Vaughan G. A. and Campàs O. (2018). Physical control of tissue morphogenesis across scales. Curr Opin Genet Dev. 51: 111 119 119. 10.1016/j.gde .2018.09.002 PMC6298228 30390520

[5] Chan C. J., Heisenberg C. P. and Hiiragi T. (2017). Coordination of Morphogenesis and Cell-Fate Specification in Development. Curr Biol. 27(18): R1024–R1035. 10.1016/j.cub .2017.07.010

[6] Schindelin J., Arganda-Carreras I., Frise E., Kaynig V., Longair M., Pietzsch T., Preibisch S., Rueden C., Saalfeld S., Schmid B., .(2012). Fiji: an open-source platform for biological-image analysis. Nat Methods. 9(7): 676–682. 10.1038/nmeth.2019 PMC3855844 22743772

[7] Bankhead P., Loughrey M. B., Fernández J. A., Dombrowski Y., McArt D. G., Dunne P. D., McQuaid S., Gray R. T., Murray L. J., Coleman H. G., .(2017). QuPath: Open source software for digital pathology image analysis. Sci Rep. 7(1): e1038/s41598–017–17204–5. 10.1038/s41598-017-17204-5 PMC5715110 29203879

[8] Weigert M., Schmidt U., Haase R., Sugawara K. and Myers G. (2020). Star-convex Polyhedra for 3D Object Detection and Segmentation in Microscopy. 2020 IEEE Winter Conference on Applications of Computer Vision(WACV). 3655–3662. 10.1109/wacv45572.2020 .9093435

[9] Stringer C., Wang T., Michaelos M. and Pachitariu M. (2020). Cellpose: a generalist algorithm for cellular segmentation. Nat Methods. 18(1): 100 106 106. 10.1038/s41592-020-01018-x 33318659

[10] van der Walt S., Schönberger J. L., Nunez-Iglesias J., Boulogne F., Warner J. D., Yager N., Gouillart E. and Yu T. (2014). scikit-image: image processing in Python. PeerJ. 2: e453. https://doi.org/10.7717/peerj.453 PMC4081273 25024921

[11] Virtanen P., Gommers R., Oliphant T. E., Haberland M., Reddy T., Cournapeau D., Burovski E., Peterson P., Weckesser W., Bright J., .(2020). SciPy 1.0: fundamental algorithms for scientific computing in Python. Nat Methods. 17(3): 261 272 272. 10.1038/s41592-019-0686-2 PMC7056644 32015543

[12] Sofroniew N., Lambert T., Bokota G., Nunez-Iglesias J., Sobolewski P., Sweet A, .(2025). napari: a multi-dimensional image viewer for Python. Zenodo. https://doi.org/10.5281/zenodo.15314358

[13] Gros A., Vanaret J., Dunsing-Eichenauer V., Rostan A., Roudot P., Lenne P. F., Guignard L. and Tlili S. (2026). A quantitative pipeline for whole-mount deep imaging and analysis of multi-layered organoids across scales. eLife. 14: e107154. https://doi.org/10.7554/elife.107154 PMC13197165 42172063

[14] Pachitariu M., Rariden M. and Stringer C. (2025). Cellpose-SAM: superhuman generalization for cellular segmentation. bioRxiv. e651001. 10.1101/2025.04 .28.651001

[15] napari clusters plotter- napari Plugin- Laura Zigutyte, Ryan Savill, Marcelo Zoccoler, Thorsten Wagner, Robert Haase. Available from: https://napari-hub.org/plugins/../plugins/napari-clusters-plotter.html

[16] Kolker J., Fischer L., Menzel A. M. and Löwen H. (2022). Elastic Deformations of Spherical Core-Shell Systems Under an Equatorial Load. J Elast. 150(1): 77 89 89. 10.1007/s10659-022-09897-1

[17] Tlili S., Durande M., Gay C., Ladoux B., Graner F. and Delanoë-Ayari H. (2020). Migrating Epithelial Monolayer Flows Like a Maxwell Viscoelastic Liquid. Phys Rev Lett. 125(8): e088102. 10.1103/physrevlett.125 .088102 32909763

[18] Moos F., Suppinger S., de Medeiros G., Oost K. C., Boni A., Rémy C., Weevers S. L., Tsiairis C., Strnad P., Liberali P., .(2024). Open-top multisample dual-view light-sheet microscope for live imaging of large multicellular systems. Nat Methods. 21(5): 798 803 803. 10.1038/s41592-024-02213-w PMC11093739 38509326

[19] Li L., Wu L., Chen A., Delp E. J. and Umulis D. M. (2023). 3D nuclei segmentation for multi-cellular quantification of zebrafish embryos using NISNet3D. Electronic Imaging. 35(17): 109 1 1 –109–9. 10.2352/ei.2023 .35.17.3dia-109

[20] Hashmi A., Tlili S., Perrin P., Lowndes M., Peradziryi H., Brickman J. M., Martínez Arias A. and Lenne P. F. (2022). Cell-state transitions and collective cell movement generate an endoderm-like region in gastruloids. eLife. 11: e59371. https://doi.org/10.7554/elife.59371 PMC9033300 35404233

